# Novel Drug Delivery Systems Fighting Glaucoma: Formulation Obstacles and Solutions

**DOI:** 10.3390/pharmaceutics13010028

**Published:** 2020-12-26

**Authors:** Ognjenka Rahić, Amina Tucak, Naida Omerović, Merima Sirbubalo, Lamija Hindija, Jasmina Hadžiabdić, Edina Vranić

**Affiliations:** 1 Department of Pharmaceutical Technology, Faculty of Pharmacy, University of Sarajevo, Zmaja od Bosne 8, 71000 Sarajevo, Bosnia and Herzegovina; amina.tucak@ffsa.unsa.ba (A.T.); merima.sirbubalo@ffsa.unsa.ba (M.S.); lamija.hindija@ffsa.unsa.ba (L.H.); jasmina.hadziabdic@ffsa.unsa.ba (J.H.); 2 Department of Clinical Pharmacy, Faculty of Pharmacy, University of Sarajevo, Zmaja od Bosne 8, 71000 Sarajevo, Bosnia and Herzegovina; naida.omerovic@ffsa.unsa.ba

**Keywords:** glaucoma, novel ocular drug delivery systems, formulation

## Abstract

Glaucoma is considered to be one of the biggest health problems in the world. It is the main cause of preventable blindness due to its asymptomatic nature in the early stages on the one hand and patients’ non-adherence on the other. There are several approaches in glaucoma treatment, whereby this has to be individually designed for each patient. The first-line treatment is medication therapy. However, taking into account numerous disadvantages of conventional ophthalmic dosage forms, intensive work has been carried out on the development of novel drug delivery systems for glaucoma. This review aims to provide an overview of formulation solutions and strategies in the development of in situ gel systems, nanosystems, ocular inserts, contact lenses, collagen corneal shields, ocular implants, microneedles, and iontophoretic devices. The results of studies confirming the effectiveness of the aforementioned drug delivery systems were also briefly presented.

## 1. Introduction

### Glaucoma

Glaucoma is a chronic disease that affected approximately 60.5 million people worldwide in 2010 [1]. This number is expected to rise approximately to 76.0 million by 2020 and 112.0 million by 2040 [2,3]. Glaucoma is the second most common eye disease after cataracts [4]. It is known as the “silent thief of vision” because its symptoms are usually only felt in the late stages of the disease when the visual field and vision are seriously impaired [5]. Yet, it is the most common cause of vision loss internationally [2,6,7].

**Figure 1:**
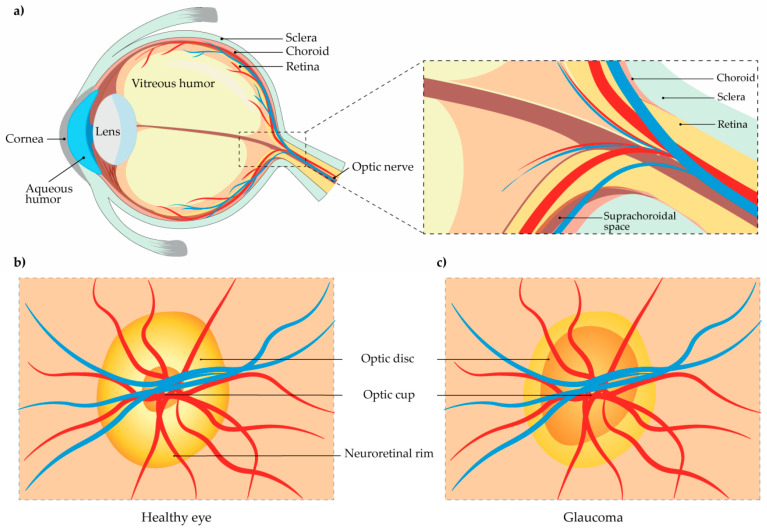
(**a**) Morphology of the eye; (**b**) Optical nerve head in a healthy eye; (**c**) The optical nerve head in glaucoma is characterized by vertical elongation of the cup and loss of the neuroretinal rim.

Glaucoma is a neurodegenerative disorder of the optic nerve. It is a form of optic neuropathy, characterized by damage of the optic disc, the place where the optic nerve and blood vessels enter the retina (Figure 1a) [8]. The retina is a thin layer on the rear eye part that collects light and it consists of neuronal and non-neuronal parts. One of five types of retinal neurons are ganglion cells [9]. These are unique retinal cells, which can produce action potentials that are transmitted to the brain through the optic nerve, thus enabling vision. Of all retinal cells, only ganglion cells, in particular their axons, are affected by glaucomatous changes. This sensitivity is because: (1) a part of ganglion cell axons, between the retina and the lamina cribrosa, is not myelinated, and (2) ganglion cell axons are very long and susceptible to numerous disorders [10]. Although glaucomatous havoc affects the retinal ganglion cell stroma, photoreceptors, the lateral geniculate body, and the visual cortex, the main reason for vision loss is the impairment of retinal ganglion cell axons within the *lamina cribrosa* of the optic nerve head [11].

The change of the optic disc, known as “cupping of the optic disc” is its vertical elongation and is accompanied by the loss of the neuroretinal rim, which can be visualized on the superior and inferior poles of the optic disc (Figure 1c) [3,12].

Most frequently, glaucomatous changes of the optic disc occur as a result of elevated intraocular pressure (IOP) [3,13,14]. However, elevated IOP on its own is not proof of glaucoma’s existence. On the other hand, it has been proven that lowering IOP in ocular hypertension delays or even stops changes in optic nerve axons [13,14].

Open-angle glaucoma is a threat called “silent thief” [5]. Another type of glaucoma, denoted as “angle-closure glaucoma”, is characterized by an increase in IOP as a consequence of a physical obstruction of the trabecular meshwork by the iris [15]. Apart from these, there is congenital or childhood glaucoma, which is a result of inadequate development of the aqueous outflow system. It may be a surprise, but there is also normal or low tension glaucoma, with normal IOP levels, but it is believed that vascular and genetic factors influence its occurrence [10,16]. There is also drug-induced glaucoma, secondary glaucoma that is induced by topical and systemic medications, especially corticosteroids [10,16]. There are types of glaucoma associated with other systemic eye diseases and conditions, such as pigmentary glaucoma, and the list continues [16].

## 2. Glaucoma Treatment

### 2.1. IOP Lowering Agents

The treatment of glaucoma depends on several factors that are related to a patient or his environment. The outcome of these factors’ action varies from person to person, and the treatment must be individualized for each patient. It is very important to notice that there are many cases of vision loss in patients with glaucoma because of non-adherence. Therefore, there are several approaches to glaucoma treatment such as laser therapy, incisional surgery, or medication use. The first-line treatment is mostly medication therapy. The failure of this therapy implies the application of other methods [17].

The IOP is currently the only known modifiable risk factor for glaucoma. It has been proven that reducing IOP can slow down the deterioration of the visual field, and thus prevent the development and progression of glaucoma [18,19,20]. Having this in mind, the therapy aims to decrease IOP to an individual target level at which further progression of glaucomatous optic nerve damage is unlikely. The target IOP level for a particular eye is estimated based on the pre-treated IOP level, the severity of the damage, present risk factors for progression, life expectancy, and the potential for side effects of the treatment. In general, the aim is to reduce IOP by 20–50%. The more the optic nerve is damaged, and more risk factors are present, the lower the target IOP level is. It should be periodically re-analyzed to assess whether the optic nerve damage is stable or progressing [3].

Briefly, the groups of medications used in the treatment of glaucoma are [17,21,22]:

- prostaglandin analogs (PAs)—latanoprost, travoprost, bimatoprost, unoprostone, latanoprostene bunod;
- beta-adrenergic blockers (BBs)—timolol, betaxolol, levobunolol, metipranolol, carteolol;
- adrenergic agonists (AAs)—clonidine, apraclonidine, brimonidine;
- carbonic anhydrase inhibitors (CAIs)—brinzolamide, dorzolamide;
- miotics—pilocarpine, carbachol, acetylcholine, demecarium bromide, echothiopate iodide;
- rho-kinase inhibitors (RKIs)—netarsudil.

Their basic task is to lower IOP, whether by increasing the aqueous humor flow or by reducing its production [17]. A meta-analysis showed that prostaglandins lower IOP by 28–33%, while slightly less reduction is achieved with BBs. The reduction achieved with AAs and CAIs is in the range of 15–20% [23]. Although the choice of therapy depends on cost, side effects, and dosing frequency, generally, PAs are the first-line medical treatment [3].

### 2.2. Wound Modulating Agents

If medication therapy, even at the highest doses or in case of causing significant side-effects, fails to control glaucoma progression, the second-choice treatment is laser therapy, either argon laser trabeculoplasty (ALT) or selective laser trabeculoplasty (SLT). SLT has been accepted as a treatment option as it has proven to be more effective and safer than ALT [24,25,26,27]. Nevertheless, it has been found that this treatment only delays the surgical intervention, as it is not much more successful than medical therapy and carries a certain risk [28]. If two of the above treatments fail, surgery may be considered to reduce the lower IOP. Various surgical options include glaucoma filtration surgery (trabeculectomy), glaucoma tube surgeries, and more recent minimally invasive glaucoma surgeries (MIGS) [29,30,31,32]. Filtration surgery creates a channel through which the aqueous humor can be drained through bleb formation [22,30,33]. However, unlike all other surgical procedures, filtration surgery does not seek a scar formation. In fact, when a scar is formed, this operation is considered unsuccessful [22,34].

**Figure 2:**
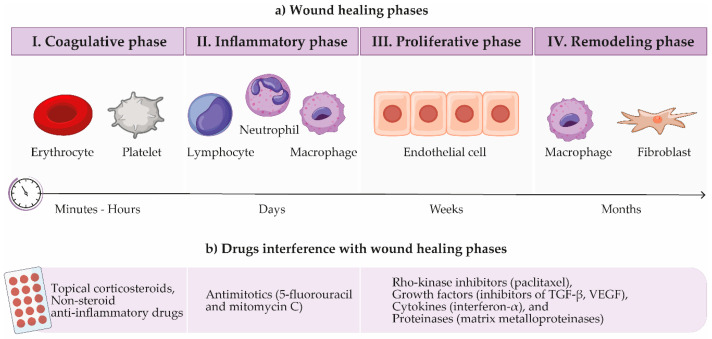
Schematic overview of the process of wound healing (**a**) and drugs that interfere with these phases (**b**). Note: TGF-β: transforming growth factor-β; VEGF: vascular endothelial growth factor.

Physical injuries to tissues, whether caused by surgery, chemical agents, or radiation, cause a series of overlapping events that lead to wound healing. It is a process that proceeds in four phases: coagulative, inflammatory, proliferative, and remodeling (Figure 2) [34,35,36]. The coagulative phase takes place immediately after surgery, as the body reacts throughout the hemostasis, leading to the formation of blood and fibrin cloths to reduce blood loss [33,35]. Activated platelets lead to the release of various chemicals and growth factors, such as platelet-activating factor, platelet-derived growth factor (PDGF), vascular endothelial growth factor (VEGF), and cytokines and chemokines including interleukin-1 (IL-1) and IL-8, transforming growth factor (TGF-β1 and β2), etc. In the inflammatory phase, neutrophils and activated macrophages release TGF-β, PDGF, and fibroblast growth factor (FGF), just as T-lymphocytes do. These growth factors contribute significantly to the wound healing process. Fibroblasts play the leading role in the proliferative phase, being recruited and activated by two key profibrogenic cytokines PDGF and TGF-β. Fibroblasts lead to angiogenesis, followed by tissue remodeling under the orchestration of enzymes such as matrix metalloproteinases (MMPs) [33,37].

Scar formation in and around the filtration bleb area is the main reason for surgery failure. Theoretically, the chances of success of the operation can therefore be increased by modulating the wound healing process by interfering with various cellular and humoral factors involved in this process [38]. Firstly, the control of wound healing can be achieved by avoiding unnecessary tissue trauma during the operation itself. Secondly, various pharmacological agents can be used to modulate certain phases of the process [29,34].

Interference with the inflammatory phase can be achieved with anti-inflammatory agents, such as topical corticosteroids and non-steroid anti-inflammatory drugs. Corticosteroids ultimately reduce fibroblastic activity and fibrosis in wound healing [34], by downregulating the expression of inflammatory cytokines, chemokines, and MMPs [39,40]. Since their tissue penetration depends on lipophilicity, lipid-soluble corticosteroids would be a better choice for controlling intraocular inflammation. The highest potency in reducing inflammation thus shows dexamethasone, followed by methylprednisolone, prednisone, and finally hydrocortisone [34].

The main obstacle in formulating dexamethasone for ocular delivery is its short residence time due to its short aqueous half-life [41], systemic side-effects [22], and the challenge of prolonging its release from the drug delivery system to ensure patient adherence to treatment. Today, the modulation of the proliferative phase of the wound healing process is mainly achieved with antimitotics, such as 5-fluorouracil (5-FU) and mitomycin C (MMC), which have significantly improved the success rate of glaucoma surgery in recent decades. Both agents are very effective chemotherapeutics with antiproliferative properties, but carry the risk of vision-threatening complications [29,33]. 5-FU antagonizes pyrimidine metabolism, which ultimately leads to inhibition of DNA synthesis and cell death [42]. However, MMC can interfere with any phase of the cell cycle, causing DNA crosslinking and thus inhibiting DNA replication, mitosis, and protein synthesis [34]. MMC inhibits fibroblast proliferation more effectively and permanently than 5-FU [43,44,45,46] but is associated with higher rates of complications such as thin avascular blebs that can lead to hypotony and endophthalmus [47]. Hypotony is a condition in which IOP falls below 5 mmHg according to World Glaucoma Association guidelines [48], although Abbas et al. have proposed a revised definition of “IOP ≤ 7 mmHg associated with choroidal detachment or maculopathy” based on the results of their study [49]. Hypotony can occur with or without sequelae, which may include shallow or completely flat anterior chamber, iridocorneal touch, choroidal detachment, or hypotony maculopathy [48,50].

Two obstacles need to be resolved when formulating antimitotics and that are related to the way they are administered: one possibility of administering antimitoics is during surgery when a small sponge of antimitotic is placed on the sclera, which is inaccurate, the dosage of drug cannot be monitored and absorption by target tissue is not guaranteed [51], the other option is performed postoperatively when a certain amount of 5-FU is injected into the bleb site daily for the first two weeks after surgery [52]. This approach is effective but can be very painful and traumatic for the patient, not to mention possible ocular toxic effects in the event of drug leakage from the injection site [53]. On the other hand, these problems can be solved by using MMC, since MMC is recognized as 100 times more effective than 5-FU and is suitable as a single-use adjunct in filtration surgery due to its prolonged antifibrotic effects [54].

## 3. Novel Drug Delivery Systems

A major problem in glaucoma treatment, as in other chronic diseases, is patients’ non-adherence. Although blindness caused by glaucoma can be avoided if glaucoma is diagnosed and correctly treated in the early stages, numerous studies have shown the problem of intermittent therapy [55,56,57,58,59,60]. A study conducted by Tsai et al. [55] identified 71 reasons for non-adherence, which were divided into four groups: situational/environmental factors, medication regimen, patents’ factors, and provider’s factors. The results of a survey lead by Newman-Casey et al. [60] showed that patients’ forgetfulness was the most common reason for poor adherence to treatment. Secondly, there was a lack of self-efficacy (missing the eye during the drop application), and then beliefs about glaucoma (patients did not believe that they could go blind), and medications (patients thought that drugs did not work).

Another issue is the drawbacks of conventional ophthalmic dosage forms. Although eye drops are easy to manufacture and account for over 90% of all commercially available ophthalmic formulations, their main flaw is poor drug bioavailability (BA) (up to 10%) [61,62,63,64]. One of the reasons for poor drug BA is the limited retention capacity of the cul-de-sac (usually 7–10 µL, maximum 50 µL) [61,65], followed by rapid drainage caused by gravity or through the nasolacrimal duct [62].

To improve patients’ adherence and eliminate the limitations of conventional glaucoma therapy, intensive work has been done regarding the development of new drug delivery systems. The development of novel drug delivery systems (NODDS) goes in two parallel directions [66]:

1. extending a drug’s contact time with the eye surface, and
2. slowing down its elimination.

Sustained release delivery systems, whether in the form of gel drops or implants, inserts, etc., are a promising approach.

### 3.1. In Situ Gel Systems

One of the most successful approaches to solving problems with conventional ophthalmic formulations is to increase their viscosity by using different gelling polymers [66,67,68]. Since too viscous formulations can cause foreign body sensation in the eye and blurred vision, it is essential to determine the optimal viscosity and rheological profile of these formulations [69].

Studies have shown that pseudoplastic fluid is the desired rheological behavior of ocular gel formulations in order not to interfere with blinking. These formulations have high viscosity at low shear rates and low viscosity at high shear rates [70,71,72].

**Figure 3:**
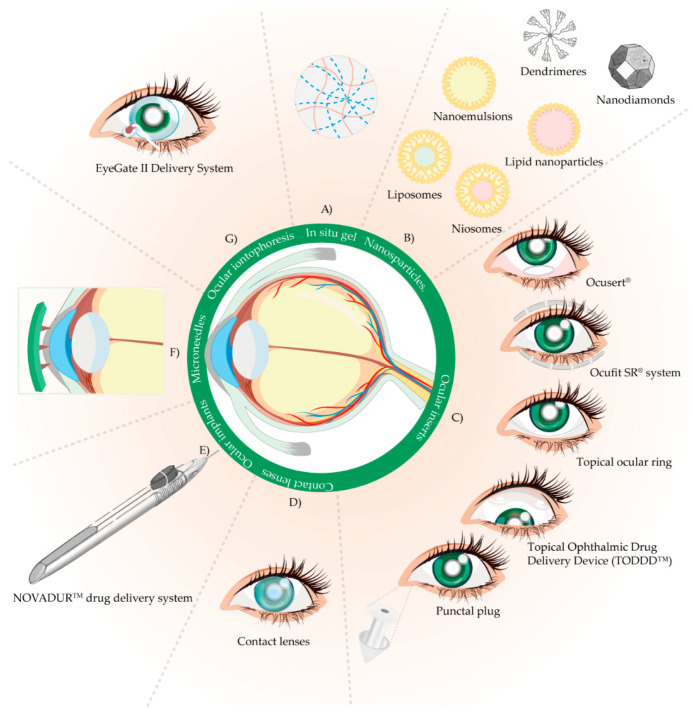
Overview of drug delivery devices for glaucoma treatment. (**A**) In situ gel systems, (**B**) Nanoparticles: liposomes, niosomes, nanoparticles, lipid nanoparticles, dendrimers, nanodiamonds, (**C**) Ocular inserts: Ocusert^®^, Ocufit SR^®^ system, topical ocular ring, Topical Ophthalmic Drug Delivery Device (TODDD^TM^), punctal plug, (**D**) Contact lenses, (**E**) Ocular implants: Novadur^TM^ drug delivery system, (**F**) Microneedles, (**G**) Ocular iontophoresis: EyeGate II delivery system.

The development of in situ gel systems (IGS) or “sol-gel” systems attracted a lot of attention, especially when it comes to sustained drug release (Figure 3A). The reason for their specific behavior is the presence of stimuli-responsive or “smart” polymers, which undergo remarkable physicochemical changes as a result of small changes in their surroundings [62,73].

Because of their specific physicochemical properties, IGS exists in the form of viscous liquid, so they can be administered like conventional eye drops, which is advantageous considering the ease of administration and safety. However, as soon as they reach the eye surface, “smart” polymers exhibit a transition to a gel state, because of the presence of certain stimuli or impulses. These stimuli can be controlled by the electric or magnetic field, ultrasound or light, and pH, temperature, or ionic alteration, as well as the presence of certain enzymes or antigens [74,75,76].

#### 3.1.1. Temperature-Sensitive IGS

Thermoresponsive IGS are the oldest, but still, the most commonly used IGS for ocular drug delivery. It is recommended that IGS are transferred to a gel state above the room temperature, preferably at the pre-corneal temperature. On the contrary, developed IGS have a gel transition temperature of 35 °C, which corresponds to the pre-corneal temperature [77].

Here, we present Poloxamers^®^, xyloglucan, and poly(*N*-isopropyl acrylamide) (PNIPAAm) as commonly used thermosensitive polymers in ophthalmic formulations [62,64,65].

##### Poloxamers^®^

Poloxamers^®^ are polymers that consist of three blocks of copolymers: poly(ethylene oxide)-poly(propylene oxide)-poly(ethylene oxide) (PEO-PPO-PEO). They are characterized by amphiphilicity, which is a result of the presence of certain functional groups. They contain hydrophilic groups of ethylene oxide and hydrophobic groups of propylene oxide. At the body temperature, they are transformed into a gel state at a concentration above 15%. Mechanisms that enable their transition to a gel state are polymer desolvation, increased micelle aggregation, and increased entanglement of the polymer network structure [65,78]. Other trade names under which they are available are Pluronics^®^ and Tetronics^®^ [78].

In ophthalmic formulations, Pluronic^®^F-127 (PF-127) or Poloxamer^®^407 (P407) is frequently used. After temperature-induced conversion, this polymer results in a clear, colorless, and transparent gel. The gel transition takes place in formulations containing 20–30% PF-127, which is a high concentration and can be irritable to the eye [62,78]. Apart from that, when Gupta and Samanta investigated the possibility of developing an IGS formulation of forskolin [79], a diterpenoid isolated from plant *Caleus forskohlii*, that lowers IOP in animals and humans by increasing aqueous humor outflow [80], they proved that P407 in a concentration above 25% forms a stiff gel at low temperature, causing difficulties in dropping the solution in the eye. So, they prepared formulations containing 18, 20, 22, and 25% P407, adding sodium chloride for isotonicity and benzalkonium chloride as a preservative and a corneal penetration enhancer for forskolin. They proved that the optimal formulation was the one containing 22% P407, with sustained drug release of 4 h and efficacy of lowering IOP for 12 h in New Zealand albino rabbits. No side effects were observed when applying this formulation, and the measurable forskolin content in tear fluid was maintained for 4 h compared to 0.5 h with conventional drops, implying that the corneal residence time was prolonged [79].

To overcome these disadvantages and reduce the PF-127 concentration, intensive work has been done regarding the development of combined IGS, which contain a combination of polymers with different transition triggers. Another approach is the combination of PF-127 with viscosity-enhancing substances, such as hydroxypropyl methylcellulose (HPMC) or methylcellulose (MC), as well as isotonic substances, such as mannitol or sodium chloride [62,78].

El-Kamel et al. investigated the possibility of developing the formulation of timolol maleate that is more acceptable to the eye and ensures sustained drug release. They determined rheological profiles of formulations with three different concentrations of PF-127: 15, 20, and 25%, namely. Then, to the formulation containing 15% PF-127 they added HPMC, MC, and carboxymethylcellulose sodium (CMC Na), as viscosity enhancers. Based on in vitro and in vivo results, they concluded that the formulation containing 15% PF-127 and 3% MC had the slowest drug release, with a cumulative release of just over 60% within 4 h, and thus the greatest potential to increase drug BA (by 2.4 times). This could be due to the inverted temperature behavior of both polymers, as they tend to gel when heated and melt when cooled. No adverse effects have been noted [70].

Darwhekar et al. prepared different formulations of dorzolamide hydrochloride and timolol maleate with PF-127 at concentrations of 15% and 20% and the addition of HPMC (0.5%, 1%, and 1.5%). Results showed that the formulation containing 15% PF-127 and 1% HPMC had optimal physiochemical and permeability properties, sustained in vitro release of 8 h compared to 1.5 h with conventional eyedrops. Moreover, this formulation was proven to be stable for 2 months, but no in vivo tests were performed, so the adverse effects remained unknown [81]. Similar results were obtained in a study by Geethalaksmi et al. They proved that the same concentration ratio of PF-127 and HPMC resulted in the optimal formulation of betaxolol chloride, with sustained drug release of 7 h. The formulation proved to be in vivo non-irritating to the eyes, and although the authors claimed that the formulation provided a longer pre-corneal residence time and thus the possibility to improve drug BA, there were no additional in vivo tests to support these claims [72]. Avinash et al. investigated formulations of clonidine hydrochloride containing different amounts of P407 and HPMC K1M. They concluded that the optimal formulation, with a sustained drug release of 6 h, contained 17% P407 and 0.45% HPMC K1M and claimed that the administration was pleasant, safe, and effective, although no in vivo tests were provided to support this [82]. Panchal et al. concluded that the best formulation of betaxolol hydrochloride contained 20% P407 and 1.5% HPMC. This formulation sustained drug release in vitro for 8 h. No in vivo tests were performed [83].

Betaxolol hydrochloride was a drug of choice in a study by Huang et al. They proved that the best performing formulation was the one consisting of 22% P407 and 3.5% P188, with the addition of polycarbophil to increase viscosity. The formulation showed a burst release of approximately 70% during the first 3 h of in vitro release tests, whereupon the release of the drug was extended to 8 h. The formulation proved in vivo to be non-irritating to the eye, while in vivo pharmacokinetics tests showed higher AUC in aqueous compared to conventional eye drops implying the possibility of improved drug BA. IOP lowering effect lasted for 12 h [84]. Lad and Bajaj formulated brinzolamide with 22% P338 and added either HPMC K4M or Carbopol 974 to increase viscosity. The formulation that sustained drug release for 24 h was composed of 22% P338 and 1% HPMC K4M. Ex vivo studies showed 85–88% of the drug permeated through the cornea in 24 h, while 10–13% of it was retained on the cornea, indicating prolonged release. Apart from that, the results showed that the formulation was non-irritant to the eye but the IOP lowering effect was not tested [85].

In a study by Alkholief et al., efforts were made to develop the optimal formulation of dipivefrin hydrochloride, a prodrug of epinephrine used in the treatment of open-angle glaucoma [86]. Formulations were prepared with Poloxamers^®^ (P407 and P188) as gelling polymers and Carbopol^®^ 934 as a viscosity enhancer. Based on the results of in vitro and in vivo tests, the optimal formulation had a suitable rheological profile, the longest pre-corneal retention of 2 h as determined in vivo, sustained drug release for 8 h, and pharmacokinetic parameters that could lead to improved drug BA, compared with the conventional dipivefrin eye drops. Furthermore, in vivo in New Zealand white rabbits demonstrated a 12 h-lasting efficacy in lowering IOP. It contained 20% P407, 5% P108, and 0.15% Carbopol [87].

##### Xyloglucan

Various polysaccharides have the potential to be used in ophthalmic formulations, such as xyloglucan, gellan gum (GG), xanthan gum, polygalacturonic acid, etc. The advantages of polysaccharides over synthetic polymers include the ease of extraction from natural resources, a variety of properties, low production costs, non-toxicity, and biocompatibility [88]. Xyloglucan, derived from tamarind seeds, has a significant swelling capacity, which is important for the initiation of bioadhesion. Furthermore, its structural resemblance to endogenous mucin enables longer adhesion to the eye surface and thus a sustained drug release [89]. The fact that xyloglucan can form macromolecular ionic complexes with a drug, which means that a drug can show its effects longer than conventional ophthalmic solutions, speaks for its role in improving drug BA [62]. All these xyloglucan properties were the reason why it has been included in studies that will solve obstacles of drug release prolongation and BA improvement in ocular topical formulations.

Burgalassi et al. incorporated timolol maleate into a xyloglucan-based gel system. They compared its rheological properties and therapeutic effect with those of conventional eye drops and IGS of timolol maleate in GG, which were two reference formulations. The tests were performed on Dutch-belted, pigmented rabbits for pre-ocular (in tear fluid), ocular (in ocular fluids and tissues), and systemic absorption studies (in plasma). During ocular absorption studies, at least 6 animals (12 eyes) were used at each time point. At the time points, 10, 30, 60, 120,180, and 240 min after application of the tested formulations rabbits were killed. On the other hand, animals were not sacrificed for pre-ocular and systemic absorption studies, since the samples were taken from the lower marginal tear strip and ear marginal vein, respectively. In the IOP study, six groups of normotensive rabbits with at least 10 animals per group were tested, so that two rabbit groups were used for each formulation. Formulations were administered once and IOP was measured every 30 min for the first hour, hourly for the next 8 h, and every two hours for the period of 15–24 h after administration. The results showed that xyloglucan-based IGS containing 2% xyloglucan, despite its comparatively lower viscosity than reference IGS, ensured high timolol concentrations in the eye while minimizing its systemic absorption. This was very important as timolol has serious systemic side effects and the main obstacle in its formulation is to reduce its systemic absorption while prolonging its ocular residence time. Conventional eye drops and xyloglucan-based IGS lead to a sharp IOP drop starting 30 min after administration, with a maximum decrease of 5.33 and 5.25 mmHg for conventional eye drops and xyloglucan-based IGS, respectively after one hour. On the other hand, GG-based IGS lead to a slow IOP drop, with a maximum decrease of 4.06 mmHg after 8 h and then a rapid IOP increase to basal values. Conversely, xyloglucan-based IGS led to a long-lasting hypotensive effect of the IOP decrease between 2.2 and 3.1 mmHg up to 19 h after administration. The IOP returned to basal values 24 h after administration. In addition, statistical analysis was performed for pharmacokinetic data only. Drug concentration in tear fluid 3 min after administration and elimination rate constants from the tear fluid were significantly different (*p* < 0.05) for both IGS formulations compared to conventional eye drops. Significant differences (*p* < 0.05) were also observed for concentration peaks in the iris-ciliary body. GG-based and xyloglucan-based IGS AUC values were 1.5-and 1.8-fold higher, respectively compared to conventional eye drops. No side effects have been reported and no human trials have been conducted [90].

Enzyme-degraded xyloglucan was used as a gelling agent in the formulation of pilocarpine hydrochloride developed by Myjazaki et al. Compared rheological properties showed that 2% xyloglucan gel strength was equivalent to that of 25% PF-127. This is an advance of xyloglucan as it can be used in formulations at significantly lower concentrations than PF-127. Xyloglucan-based IGS showed sustained drug release for over 6 h. The formulation containing 1.5% xyloglucan caused miosis during a minimum of 4 h, as did one with 25% PF-127 [91].

##### Poly (N-Isopropyl Acrylamide)

The PNIPAAm is a thermosensitive polymer that is most extensively studied and used in drug delivery systems, as it is soluble in water at room temperature. Furthermore, its gelling temperature can be changed by copolymerization with, e.g., acrylic acid and PEO [74].

Hsiue et al. developed formulations based on PNIPAAm with epinephrine. They contained either linear or a combination of linear and cross-linked PNIPAAm. The administration of formulation with linear PNIPAAm resulted in six times longer IOP reduction compared with the conventional eye drops, whereas the formulation containing a combination of linear and cross-linked PNIPAAm reduced IOP eight times longer than conventional eye drops. The formulation showed no corneal cytotoxicity, as tested on New Zealand white rabbits [92].

PNIPAAm proved to be a good approach in sustaining ocular drug release, but its major disadvantage is that it produces a rigid and unpleasant film on the cornea. This is one of the formulation obstacles when working with this polymer which Cao et al. tried to solve by developing IGS with PNIPAAM-chitosan (CS) derivative and timolol maleate. Compared to conventional timolol eye drops, this IGS showed a greater IOP reduction, and the effect lasted longer, up to 12 h. The cytotoxicity test showed good ocular tolerance of this formulation. Since the pharmacokinetics test showed a higher aqueous humor concentration of timolol (11.2 ng/mL) compared to conventional eye drops (5.58 ng/mL) and AUC two times greater, it is suggested that this formulation may improve ocular BA [93].

Eye drops, based on the mixture of poly (acrylic acid-graft-*N*-isopropyl acrylamide) (PAA-graft-PNIPAAm) with PAA-co-PNIPAAm gel with epinephrine, were developed by Prasannan et al. The researchers used this polymer mixture to overcome an obstacle in the application of the formulation of single crosslinked gel, i.e., the system becomes too small and drug loss due to drainage occurs. The PAA-graft-PNIPAAm showed faster drug release, while the mixture of PAA-graft-PNIPAAm and PAA-co-PNIPAAm gel showed a sustained drug release profile. The IOP was reduced for 36 h, which represented a considerable prolongation of the effect compared with the 8 h-lasting efficacy in lowering IOP observed after the administration of traditional eye drops. Apart from that, a smooth film was formed after application, which caused no discomfort in laboratory animals. The results also showed that the kinetics of drug release from the polymeric eye drops is determined by the cross-linking density [94].

Bellotti et al. worked on lowering PNIPAAm gelling temperature by polymerization with polyethylene glycol (PEG) and the inclusion of brimonidine tartarate as an active substance. Lowering the gelling temperature is a solution to prevent gel drops to restore fluidity in cold or windy weather, and to ensure rapid gelling after administration. The formulation showed sustained drug release for 28 days, with the amount of brimonidine tartarate exceeding the minimum absorption limit specified in the literature over the entire duration of the study. In tests on human conjunctival epithelial cells, the formulation showed no cytotoxicity [95].

In a study conducted by Lai et al., optimized pilocarpine-loaded glutathione-PNIPAAm IGS effectively suppressed glaucoma progression (IOP reduction) for 14 days, whereas the use of the simple glutathione-based formulation reduced glaucoma development for three days. In 30 New Zealand white rabbits, five IOP measurements were performed by using Schiotz tonometer on each eye and the average IOP was calculated. IOP dropped to baseline values 12 h after a single administration and remained close to baseline values for 14 days. However, the authors seem to use the terms glaucoma development and glaucoma progression interchangeably, as they stated that the suppression of glaucoma progression/development can be estimated by the ability of the formulation to alleviate changes in the glaucomatous corneas, such as aberration. To evaluate this parameter, the authors used topographic maps of the cornea. Results showed a uniform green color distribution, which, according to the authors, was an indicator of low levels of corneal aberration. To support topographic maps, the authors calculated the mean keratometric K values of corneal curvature. The results showed statistically significant differences (*p* < 0.001; *n* = 6) compared to healthy or untreated glaucomatous eyes. Moreover, pilocarpine concentration in aqueous humor determined after aspiration of aqueous humor from rabbit eyes by a 30-gauge needle was eight-fold higher in IGS than in glutathione-based formulation. Formulation proved to be biocompatible as tested on Statens Seruminstitut Rabbit Cornea cells. Ocular retention studies were conducted on New Zealand white rabbits by collecting the residual hydrogel matrix by washing the ocular surface with a buffer solution. Results showed prolonged ocular retention (more than 14 days) of the developed formulation and did not cause any ocular discomfort or irritation in the rabbit eyes. Furthermore, final experiments performed 14 days after single administration used transmission electron microscopy (TEM) to examine morphological structures of the myelin of the optic nerve cross-sections. In order to perform these experiments, all rabbits were euthanized. TEM images showed relatively condensed organization of the retinal axons with uniform myelin sheaths and high axon density after single topical administration, which was in contrast to the results in the group of rabbits with glaucoma and without therapy [96].

A study carried out by Chauhan et al. provided an interesting solution to sustain the release of decorin, a novel TGF-β inhibitor, on the cornea. The novelty of this formulation was that they modified gellan gum to obtain a system that can dynamically switch between solid liquid-solid to ensure a sustained delivery with increased durability compared to typically used in situ gelation. They sheared while heating aqueous gellan gum gel in the rheometer and when the temperature reached 40 °C they added decorin and NaCl as a cross-linking agent. Bioefficiency and activity were tested in vitro and ex vivo probing cytotoxicity and healing ability. Decorin formulation was found to have good compliance with cells and effective in scarring reduction. When tested ex vivo on an organ culture model for ocular healing, it was found that gellan gum was inert and successfully retained decorin for therapeutic effect. Release data showed cumulative release of up to 45% over 3 h, while inhibition of collagen deposition as an indicator of scar formation continued for 12 days in vitro using primary human corneal fibroblasts. On the other hand, the ex vivo results showed stimulation of re-epithelization within two days [97].

#### 3.1.2. pH-Sensitive IGS

All pH-sensitive polymers consist of an acidic or a basic group that can either accept or releases a proton in response to changes in environmental pH values. Polymers with many ionizable groups are called polyelectrolytes [98]. The most commonly used pH-responsive polymers in ophthalmic formulations are PAA, polycarbophil, CS, and cellulose acetate phthalate (CAP) [62].

In ophthalmic formulations with high concentrations of PAA (Carbopol^®^, Carbomer^®^), the low pH value of the PAA solution could cause damage to the eye surface before being neutralized by the lacrimal fluid [61]. This obstacle was solved by partially combining PAA with HPMC or other inert, viscosity-enhancing polymers, without affecting the general rheological properties of the formulation [62].

Barse et al. developed the formulation of brimonidine tartarate containing a combination of Carbopol^®^ 974P (0.45%) and HPMC K4M (1%). The IGS provided sustained drug release for 8 h, while conventional eye drops released a drug for 2 h, as determined by in vitro release studies. Likewise, IOP was reduced by 13.38 ± 4.42% during 2 h with conventional eye drops, in contrast to the reduction of 45.71 ± 4.72% during 8 h caused by IGS, which was determined by an in vivo study on New Zealand albino rabbits using a Schiotz tonometer. All measurements were performed in triplicate and mean values were taken. Furthermore, an ex vivo transcorneal permeation study with goat eyeballs was performed, which showed drug permeation of 76.83 ± 1.6% up to 5 h after IGS application, as opposed to drug permeation of 74.12 ± 1.3% up to 1 h with conventional eye drops [99]. Pang et al. prepared brimonidine tartarate (0.05%, 0.1%, and 0.2%) IGS with Carbopol^®^ 974P (0.3%) and HPMC K4M (6%). Gels with lower concentrations of this drug (0.05% and 0.1%) significantly reduced IOP compared with the conventional eye drops (0.2%), as was demonstrated in an in vivo study on New Zealand white rabbits. IOP measurements were performed in triplicate with a Schiotz tonometer. The decrease in IOP by conventional eye drops was greater than that of IGS during the first 1.5 h. After 1.5 h, the IOP decrease for IGS was greater than for eye drops. The results also showed that IGS with the highest drug concentration (0.2%) prolonged IOP lowering over 10 h, which was significantly longer than that of eye drops (8 h). The maximum decrease in IOP was 10.24 ± 0.73 mmHg and 11.82 ± 0.44 mmHg for IGS with 0.1% and 0.2% drug, respectively. This differed significantly (*p* < 0.01) from 7.37 ± 0.38 mmHg obtained with eye drops. In vivo ocular irritation studies conducted using the Draize technique showed that IGS was non-irritant to the ocular tissues. The histological examination of the rabbit eyes showed normal and healthy ocular tissues. In vivo measured precorneal residence time showed 3 h residence time on the corneal surface and in the conjunctival sac, which was significantly longer (*p* < 0.01) than the 30 min residence time achieved with conventional eye drops. Pharmacokinetic test in rabbit plasma showed that Tmax for IGS (0.05%, 0.1% and 0.2%) was 2.00 ± 0.55 h, 2.83 ± 0.75 h and 2.67 ± 0.52 h, respectively, which was significantly longer (*p* < 0.01) than tmax 0.92 ± 0.20 h of eye drops. Cmax after IGS (0.05%, 0.1% and 0.2%) administration were 6.18 ± 2.48 ng·mL^−1^, 7.51 ± 3.37 ng·mL^−1^ and 7.75 ± 3.06 ng·mL^−1^, respectively. This was significantly less (*p* < 0.05) than 18.07 ± 7.44 ng·mL^−1^ after eye drop administration. The values for AUC_Rel (0–∞)_ in comparison to eye drop was found to be 0.49 and 0.57 for 0.05% and 0.1% IGS respectively, which is significantly different (*p* < 0.05) from that of the eye drops. And the values of AUC_Rel (0–∞)_ for the 0.2% gel was found to be 0.86, which was also slightly lower than that of eye drops. This proved that gels with lower concentrations of brimonidine tartarate were able to reduce systemic absorption and thus prevent systemic toxicity [100].

In a study by Bharath et al., Carbopol^®^ 940 (0.6%) and HPMC (0.4%) formed brimonidine tartarate-IGS with desired properties. The formulation showed prolonged release of this drug throughout 8 h and therefore extended the residence time in the eye. Furthermore, results showed that formulations did not irritate or damage the cornea, iris, and conjunctiva. Formulation did not cause ocular irritation in in vivo tests on rabbit eyes [101].

Dorzolamide hydrochloride was an active substance in IGS based on Carbopol^®^ 940 (0.1%) and HPMC F4M (0.1%) developed by Kouchak et al. This system presented the character of a pseudoplastic fluid. Both in vitro and in vivo results showed that this vehicle performed better in drug retaining formulation (8 h) in comparison with the simple dorzolamide solution. The IOP reduction caused by IGS was greater in intensity and extension than that of conventional eye drops [102].

Gupta et al. developed 0.4% Carbopol^®^/0.5% CS-based IGS with a timolol maleate. The formulation showed a sustained drug release for over 24 h, as determined in in vitro release tests. IOP was determined in an in vivo study on rabbit eyes with Schiotz tonometer and more pronounced and longer-lasting effects on IOP. IGS showed a slower onset of action followed by an intensive IOP reduction. Peak IOP reduction was reached after 7 h, which was significantly slower (*p* < 0.05, *n* = 6) compared to 4 h with liposome formulation and 1.5 h with conventional eye drops. However, the authors did not provide exact measured IOP values. The magnitude of the pharmacological response was determined by the AUC and the relative magnitude of the biological response (BR_rel_) was calculated as the ratio of AUC for formulation and AUC for aqueous solution. AUC for IGS was 60.425 ± 3.2 mmHg/h, which was significantly larger (*p* < 0.05, *n* = 6) than AUC for conventional eye drops (24.35 ± 3.5 mmHg/h) and liposome formulation (29.2 ± 2.5 mmHg/h). BR_rel_ was 2.481-folds for IGS, while for liposome formulation it was 1.199-folds. The authors claim that the IGS achieved longer contact with the corneal surface compared to liposomes and eye drops but did not provide numerical data to support their claims. They suggested that longer residence time was a reason for the reduction of systemic drainage through the nasolacrimal canal and thus lower systemic absorption. Results also showed that 2.08% of the drug was drained through the nasolacrimal canal 10 min after administration of the eye drops. On the other hand, only 0.862% of the drug was drained 2 h after IGS application. In this way, BA can be increased as well as the dosing frequency from 4 to 2 times a day. Formulation did not cause ocular irritation in in vivo tests on rabbit eyes, as claimed by authors but no accompanying data were provided [103].

This was an elegant solution, as the obstacle to working with CS is that it can convert into a hydrogel at the ocular pH value. However, the formed gel requires further cross-linking to produce a gel with sufficient mechanical stability and release a drug in a controlled manner. The structural strength of a polymer can be improved either by blending with other polymers or by its hydrophobic modification, which was solved by adding Carbopol^®^ [98].

A similar approach, but now with another polymer used Gupta et al. which developed the formulation of timolol maleate-loaded CS/HPMC-based polymer matrix to improve ocular retention. The developed formulation was non-to mild-irritating, well-tolerated, showed improved transcorneal drug permeation and extended corneal retention thanks to the viscosity and bioadhesion of CS, as well as sustained drug release for 12 h. [104].

The flexible CS hydrogel, which was co-cross-linked with β-glycerophosphate disodium salt hydrate (β-GD) and the natural cross-linking reagent genipin with timolol maleate, was investigated by Song et al. In vitro release profiles showed that cross-linking with genipin reduced the release rate of entrapped timolol maleate and released it in a sustained manner. Furthermore, the administration of timolol maleate containing CS-gelatin IGS showed a longer-lasting and more effective IOP reduction for up to 24 h compared with the conventional eye drops. Cytotoxicity tests showed that a formulation containing genipin without cross-linking had relatively low cytotoxicity but showed no cytotoxic effects when genipin was cross-linked. No ocular irritation was also observed [105].

Dexamethasone is a lipophilic drug that permeates the membranes easily on the one hand, but on the other, its hydrophobicity limits its clinical usefulness. To resolve this obstacle Kesavan et al. used hydroxypropyl-β-cyclodextrin (HP-β-CD) as a solubilizer and penetration enhancer in the development of pH-sensitive IGS. Optimized formulation contained 0.2 and 0.4% of Carbopol^®^ 980NF and NaCMC, respectively. In vitro release tests showed sustained release of dexamethasone for 12 h, which was significantly slower (*p* < 0.01) than the marketed formulation. In vivo studies on rabbits showed that those treated with pH-sensitive IGS had significantly lower inflammation [106].

#### 3.1.3. Ion-Activated IGS

The presence of different ions (Na^+^, K^+^, Ca^2+^) in tear fluid can cause a phase change in certain polymer dispersions. The anionic nature of these polymers results in an attractive interaction between a polymer and oppositely charged ions. As these attractive forces unite, they induce a conformational change in the polymer structure that leads to the sol-gel transition of a polymer dispersion. Two of the most commonly used ion-sensitive polymers in ophthalmic drug delivery are GG and sodium alginate (SA) [74].

Gelation of GG increases proportionally to the amount of monovalent or divalent cations in the lacrimal fluid. As a result, the usual, reflex tearing, which leads to the dilution of viscous solutions, further increases the viscosity of the formulation by increasing the lacrimal volume and thus the cation concentration [107].

The optimal formulation of brinzolamide, developed by Sun and Zhou, had a concentration of GG of 0.25%. In vitro release profiles showed that the release of brinzolamide from IGS had sustained characteristics. The IGS released this drug for over 12 h, compared with the conventional eye drops, which released it within 2 h. Apart from that, IGS were less irritating than commercial eye drops, as demonstrated by in vivo rabbit irritation test using the Draize method. Histological analysis was performed in order to access long-term irritation. Figures of microscopic preparations showed a satisfactory epithelium and stroma structure with little edema after administration of normal saline. IGS, on the other hand, showed slight edema. However, there was no significant difference between these two groups (*p* < 0.05). IOP lowering effect lasted 6 h and showed a different profile compared to commercial eye drops. IGS decreased IOP by 18.2% after 1 h, followed by slow a increase to 18.6 mmHg below baseline values after 6 h. On the other hand, eye drops caused a 27% decrease in IOP after 1 h, but IOP recovered quickly to baseline values after 6 h (21.2 mmHg). IOP reduction with IGS was significantly higher (*p* < 0.05) compared to conventional eye drops. IOP was measured in triplicate with a tonometer eight times (0, 0.5, 1, 2, 3, 4, 5, and 6 h) [108].

Brinzolamide was also a drug of choice for Bhalerao et al., who incorporated it into GG-based IGS, containing dimethyl sulfoxide as a cosolvent for brinzolamide and polyoxyl 35 castor oil as a surfactant that can reduce the amount of dimethyl sulfoxide without affecting the drug solubility. The IGS showed a longer retention time of 16–24 h in the eyes compared with the conventional eye drops. The IGS also appeared to be more effective in the amplitude and duration of lowering IOP with the addition of being non-irritant to the eyes [109].

Alginate is a linear co-polysaccharide consisting of mannuronic acid-(M) and guluronic acid-(G) residues connected with 1–4 glycosidic bonds. Gel formation happens probably because of the interaction of calcium ions with the G moieties. Alginate with high G content (of more than 65%) forms a gel about 10 min after administration, as determined by in vitro gelation studies. However, in vitro release studies showed that IGS sustains drug release for up to 24 h compared with the alginate with low G content. In addition, alginate with low G content showed an initial burst release of more than 20%, while those with high G content showed no burst release. The IGS with high G content containing alginate with pilocarpine reduce IOP for 10 h while the reduction with conventional eye drops was up to 3 h. The IOP was measured in triplicate in the test eye of New Zealand albino rabbits using a Model One Pneuma-Tonometer. Results showed that conventional eye drops resulted in a maximum IOP reduction of 4.4 ± 0.24 mmHg after 2 h, while IGS with high G content resulted in a maximum IOP reduction after 3 h, but no numerical data were provided for later [110]. To test its effectiveness in a combination, IGS containing 2% alginate were compared with the ones containing 14% Poloxamer^®^ and the mixture of 0.1% alginate and 14% Poloxamer^®^. The IGS containing the mixture formed stronger gels and retained drug better than IGS based on one gelling agent. Almost all pilocarpine in the simulated tear fluid (STF) was released immediately after the experiment began. Pilocarpine in 2% alginate solution released about 77% to the medium after 15 min, and almost all were released after 90 min. Due to the varying degree of gel strength, the release rate of pilocarpine in the 14% Poloxamer^®^ solution was much slower than in the 2% alginate solution. Only about 21% were released to the medium after 15 min, 53% after 60 min, and almost 100% after 4 h. The release rate in 0.1% alginate/14% Poloxamer^®^ combination was significantly lower, only about 12% released after 15 min, 34% after 60 min, 74% after 4 h, and about 90% after 6 h. Results show that the 0.1% alginate/14% Poloxamer^®^ mixture was best able to retain pilocarpine. In addition, in vivo studies were conducted on New Zealand albino rabbits to determine pupillary diameters in order to evaluate pharmacological response. The measurement was performed with a micrometer at the following times: 1, 15, 30, 45, 60, 90, 120, 150, 180, 240, 300, and 360 min. To assess the extent of the overall pharmacological response, areas under the decrease in pupil diameter were compared to time profiles in 360 min (AUC_0-360_). All measurements were performed in triplicate. In the first minute, the pharmacological responses of STF and alginate solutions were higher than those of Poloxamer^®^ and alginate/Poloxamer^®^ combination due to their weak gel strength and rapid release rate of pilocarpine. After 15 min, however, the pharmacological responses of Poloxamer^®^ and the alginate/Poloxamer^®^ combination were higher than those of STF and alginate solutions due to their sustained drug release. At 30 min, the reduction in pupil diameter of STF was lowest, with Poloxamer^®^ and alginate/Poloxamer^®^ combination showing almost the same pharmacological response. At 90 min, almost no pharmacological response was observed in STF or alginate solutions. The pharmacological response of the Poloxamer^®^ combination corresponded to that of the alginate/Poloxamer^®^ combination. However, the decrease in pupil diameter was lower for the Poloxamer^®^ combination, between 45 and 300 min. The overall miotic response of the alginate/Poloxamer^®^ combination was greatest. AUC_0-360_ results show that a 4.38-fold increase (*p* < 0.05) in total miotic response was achieved for the alginate/Poloxamer^®^ combination [111].

Numerous studies have been conducted to test the possibility of formulating IGS by combining polymers with different stimuli-sensitivities. Using different combinations not only reduces the required concentration of an individual polymer, but also strengthens the system’s responsiveness to multiple stimuli. Research also focuses on the formulation of stimuli-sensitive polymers as colloidal carrier systems, such as polymeric micelles, nanosuspensions, or lipid-based nanocarriers. This has led to the increased therapeutic efficacy and drug BA of ophthalmic drugs [61,62].

### 3.2. Nanosystems

Nanotechnology is defined as the science conducted at the nanoscale (1–100 nm) [112]. Many nanomaterials have interesting features, such as stability, non-toxicity, biocompatibility, and biodegradability, as well as electrical conductivity, and magnetic properties and their choice depends on a drug (its hydrophobicity, size, and stability), target ocular tissue, and route of administration [113,114]. Nanotechnology-based drug delivery systems have the potential to improve patients’ adherence, reduce side effects, increase efficacy, and preserve vision in patients with glaucoma [115]. Surface, size, and shape properties of nanosystems have an impact on the drug release and occurrence of side effects [116].

Natural, synthetic, or semi-synthetic polymers can be incorporated into nanoformulations used to treat glaucoma. Natural polymers can be anionic, cationic, amphipathic, or neutral. The most important anionic polymers are alginic acid, carrageenan, chondroitin sulfate, dextran sulfate, and hyaluronic acid. A type of cationic polymers is CS; collagen, fibrin, and gelatin are amphipathic, whereas neutral polymers include dextran. They all mimic the extracellular matrix but can show batch-to-batch variation, cause immunogenicity, and be susceptible to cross-contamination. On the other hand, synthetic polymers have a defined structure, easily controlled properties, and do not exert immunogenicity. They are easy to process via different methods. Because of the poorer mechanical properties of natural polymers and modest biocompatibility of synthetic polymers, combined natural–natural, natural–synthetic, and synthetic–synthetic polymers, such as collagen-acrylate, or alginate-acrylate, have been developed [117].

Cationic lipid polymers tend to interact with the negatively charged hyaluronan in the vitreous cavity. Poly(styrene) (PS) nanospheres exhibited an interaction with collagen in the sclera, which resulted in poor diffusion through the vitreous cavity. Compared with the nanoparticles with the positive zeta potential of 11.7 mV, nanoparticles with the negative zeta potential of −33.3 mV diffused more freely through the vitreous cavity. Many modifications have been developed to cope with these issues, such as masking the reactive nanoparticle surface, targeting the specific transporters or receptors on the cell surface, or nanoparticle surface modification with PEG [118]. Anionic polymer, hyaluronic acid, can enhance pharmacokinetic drug characteristics when combined with either methacrylic anhydride or adipic dihydrazide in ophthalmic nanoformulations. This functionalized hyaluronic acid was formulated into a hydrogel, which can be loaded with either an unbound drug form or drug-loaded liposomes. These are known as hyaluronic acid-based nanocomposite hydrogels, which can provide controlled drug release [119,120].

Possible local and systemic toxicity of nanoparticles should be analyzed during preclinical studies. Also, the majority of studies regarding the use of nanoparticles in glaucoma have been performed in animals. Human studies are required for the confirmation of the actual cytotoxicity, tolerability, and efficacy of nanoparticles. Some side effects that nanoparticles could cause are cellular toxicity through oxidative stress, interaction with the cell membrane, and inflammation [121]. Nanotoxicity is influenced by particle size, shape, surface, as well as molecule aggregation and concentration, or dosage regimen [122]. In vivo-like nanotoxicity studies on 3D human organs and cells will replace conventional in vitro studies [123,124]. In vitro methods assess physicochemical properties of nanomaterials, the environment-target cell, cellular uptake, and epigenetic interaction [125]. Transcriptomics and proteomics, as well as personalized toxicology, give information regarding the nanotoxicity and the interaction of nanomaterials with the microenvironment. Legislation should be carefully implemented, and researchers of various fields, such as pharmacy, medicine, technology, and biomedical engineering, should be included in further research [126].

#### 3.2.1. Nanoparticles

Different antiglaucoma drugs have been incorporated into nanoparticles (Figure 3B). Hyaluronic acid-modified CS nanoparticles can be a promising drug delivery system in the treatment of glaucoma [127].

Polymeric nanoparticles increase drug retention time precorneally, especially with CS, which is one of the natural polysaccharides with mucoadhesive properties. It has limited solubility in water and better solubility in acidic solutions [128].

Li et al. developed betaxolol-loaded CS/montmorillonite (MT) nanoparticles, size of 460 ± 0.6 nm and zeta potential of 29 ± 0.18 mV. The area under the curve (AUC) and mean residence time (MRT) were 1.99 and 1.75 times higher compared with those of the betaxolol solution, respectively. Thus, CS/MT nanoparticles increased drug BA. In vitro and in vivo studies presented prolonged precorneal retention time because of the tight contact with the negatively charged mucin in the cornea. In vitro study of the drug, the release showed a controlled release pattern: an initial burst release (mostly because of the drug adsorption on the preocular surface) was followed by a sustained drug release for 10 h. The IOP reduction of nanoparticles (4.44 mmHg) was greater compared with that of the solution (5.04 mmHg), because of the sustained drug release and longer retention on the preocular surface. Nanoparticles could make a significant IOP reduction and produce a therapeutic effect in rabbits [129].

Warsi et al. formulated dorzolamide-loaded poly(d, l-lactide-co-glycolide) (PLGA) nanoparticles, size of 129 ± 12.3 nm, with polyvinyl alcohol (PVA) and tocopheryl PEG 1000 succinate (TPGS) as emulsifiers. Transcorneal permeation was up to 2.5 times greater compared with the conventional solution. A biphasic release profile was exhibited: initial burst release (28.15–34.89% over 1 h, because of the hydrophilic drug nature, as well as the drug adsorption on the surface) was followed by a sustained drug release (79.50–91.78% over three days). Nanoparticles with TPGS showed the maximum IOP reduction of 29.12% after 4 h, and an IOP reduction of 22.8% was maintained for 20 h. Nanoparticles with PVA showed the maximum IOP reduction of 22.8% after 4 h, and an IOP reduction of 16% was maintained for 16 h, as demonstrated in an in vivo study in rabbits. Both formulations were safe and non-irritant for the ocular application. They can enhance patients’ adherence and show the potential of being developed into eye drops that would be applied once a day and be safe for long-term use [130].

Salama et al. formulated a subconjunctival injection of brinzolamide-loaded PLGA (A19 and B11) nanoparticles. Slow drug release was demonstrated in vitro, for up to 25 weeks. Polymeric PLGA nanoparticles released the drug for up to 10 days in rabbits. For A19 nanoparticles, AUC was 532.9 ± 66.1% days, MRT was 98.7 ± 2.2 days, and maximum IOP reduction was 78.4 ± 3.4%. For B11 nanoparticles, AUC was 395.0 ± 46.6% days, MRT was 83.9 ± 2.7 days, and maximum IOP reduction was 71.6 ± 2.0%. Results showed that A19 nanoparticles possessed higher values for AUC and MRT compared to those of B11 nanoparticles, because of the smaller size of A19 nanoparticles (193.00 ± 0.40 nm) compared with that of B11 nanoparticles (660.75 ± 51.61 nm). In vivo studies showed lower values for tmax (3 h) for A19 nanoparticles, compared with that of B11 nanoparticles (36 h). It seems that PLGA with a low molecular weight leads to faster polymer degradation and drug release. This lack of in vitro and in vivo correlation could be because of the enzymatic degradation of PLGA nanoparticles. A biphasic release profile was exhibited: initial burst release (because of the drug adsorption on the surface) was followed by a sustained drug release [131].

Khan et al. prepared CS-coated PLGA nanoparticles of forskolin, size of 201.56 ± 10.92 nm, and zeta potential of 10.1 ± 3.49 mV. The use of both PLGA (synthetic polymer) and CS (natural polymer) helped in achieving better permeation and mucoadhesiveness on the corneal and scleral surface. Drug release was slow, with 90% of release in 72 h. Both polymers provided a sustained drug release and reduced IOP for a longer time. The maximum effect of forskolin was produced at 8 h when IOP was 16.3 ± 0.75 mmHg, which was significantly lower than the initial value (25.2 ± 0.98 mmHg). At 24 h, IOP was 20.6 ± 1.03 mmHg, as determined in an in vivo study in rabbits. The sustained effect was achieved because of the presence of CS as a coating layer. An increase in PVA concentration led to a decrease in nanoparticle size, because of the rise in the viscosity of the aqueous phase and the development of a stable emulsion. On the contrary, an increase in PLGA concentration led to a smaller increase in nanoparticle size. Unbalanced dispersibility in the aqueous phase appeared with the molecule aggregation, thus increasing nanoparticle size. An increase in nanoparticle size was found with the gradual rise in CS concentration because of the enhancement of viscosity in the CS-PVA solution. Nanoparticle size decreased because of their non-aggregation and stability in the continuous phase. Forskolin-loaded CS-PLGA nanoparticles can be successfully utilized as an alternative to conventional dosage forms such as eye drops for the treatment of glaucoma, as they were non-irritant and well-tolerated, without any signs of inflammation [132].

Bhagav et al. created brimonidine tartarate-loaded Eudragit^®^ nanoparticles to examine its prolonged release in rabbits’ eyes. Eudragit^®^ nanoparticles are inert polymeric co-polymers created for the entrapment of lipophilic drugs. Formulations with a higher PVA concentration resulted in higher initial burst release, because of reduced nanoparticle size and increased effective surface area, whereas formulations with a lower PVA concentration resulted in a slower drug release. The lower PVA concentration showed a bigger nanoparticle size, thus prolonged drug release. No signs of ocular irritation or toxicity were shown, whereas IOP reduction lasted longer compared with the conventional eye drops. The maximum IOP reduction and AUC was 7.77 mmHg and 204.93 h mmHg (BENP-D30), 7.97 mmHg and 151.73 h mmHg (BENP-IP4), 7.71 mmHg and 136.33 h mmHg (BENP-PF20) and 7.6 mmHg and 268.09 h mmHg (BENP-1:1(150)), as determined in an in vivo study in rabbits. These formulations were well-tolerated and there were no signs of irritation or inflammation. Patients’ adherence can be increased with these dosage forms because of the reduction of the administration frequency in the treatment of glaucoma [133]. Brimonidine tartarate-filled CS nanoparticles, size of 270–370 nm and zeta potential of 26.2–29.8 mV, which indicates that free cationic groups remained on the surface, were prepared by ionotropic gelation after the addition of sodium tripolyphosphate (TPP). As the concentration of TPP increased, particle size increased, and viscosity of the formulation decreased. Drug release followed a biphasic pattern, characterized by initial burst release (40–45% in min, because of an unentrapped drug in the formulation and the fact that the large surface area can adsorb the drug) followed by a sustained drug release for 4 h, because of the slow drug diffusion through the matrix of CS nanoparticles. This may reduce dosing frequency by providing delayed drug release. The IOP reduction started within 30 min (−4.80 ± 1.57 mmHg), showed a peak at 5 h (–10.10 ± 1.87 mmHg), and a significant effect was noticed for up to 8 h (–1.17 ± 1.01 mmHg), as determined in an in vivo study in rabbits. Brimonidine tartarate-loaded CS nanoparticles were proven to be safe for the ocular application. They can reduce the application frequency because of the sustained drug release in the treatment of glaucoma [134].

Positively charged pilocarpine hydrochloride-filled polymeric and lipid nanoparticles, size of 73–3179 nm and zeta potential of 42.77–47.5 mV, prepared by quasi-emulsion solvent evaporation, are risky for use because of the occurrence of side effects. Reduced nanoparticle size and increased surface area led to an increase in the amount of the drug conducted with aqueous media [135]. Pilocarpine-loaded nanoparticles of 294 nm prepared by dropping method increased miotic response to 40%. CS dropped into the Carbopol solution led to the formation of nanoparticles with a CS core and CS/Carbopol membrane. After addition, pilocarpine was entrapped between the core and membrane. Zeta potential was 73.46 mV in CS and 50.66 mV in CS/Carbopol mixture. When pilocarpine was incorporated, zeta potential increased from 50.66 to 55.78 mV, because pilocarpine has a positive charge. Positive zeta potential suggested that the positive CS or pilocarpine molecules, or both, were distributed nearer the surface than those of the negative Carbopol molecules. Nanoparticles showed an initial burst release followed by a sustained drug release for 24 h. The AUC was 751.6, indicating that this nanoformulation is one of the best delivery systems for pilocarpine [136]. Polymers like gelatin are gaining appreciation in ocular drug delivery because of their safety and availability [137]. Liao et al. prepared gelatin-coated mesoporous silica nanoparticles of pilocarpine, size of 50 nm. They showed high drug release (50%) lasting up to 36 days in vitro. In vivo data in rabbits showed that nanoparticles were able to reduce IOP for 21 days. There is an important role of particle size in blocking the filtration of the trabecular meshwork, which could account for its loss in the anterior chamber. Nanoparticles presented a long-lasting drug release and a successful IOP reduction [138].

The formulation of methazolamide-bound calcium phosphate nanoparticles, size of 256.4 ± 31.1 nm and zeta potential of −30.4 ± 2.2 mV, prolonged the duration of reduced IOP (6–18 h), which was useful in the local treatment of glaucoma. Nanoparticles were prepared by forming an inorganic core of calcium phosphate on which methazolamide was adsorbed. After 4 h, 99.4% of methazolamide was released. This could be because of the crossing cell membrane activity [139].

Lipids are easily available from natural sources, but the negative charge of solid lipid nanoparticles (SLNs) presents a problem for drug penetration and absorption on the corneal surface. Thus, SLNs were coated with cationic polymers to increase corneal drug absorption. CS-coated methazolamide-loaded SLNs, size of 188.2–191.6 nm and zeta potential of −8.1 to −10.7 mV, are a form of more intensive treatment of glaucoma. SLNs were prepared by a modified emulsion-solvent evaporation method. Methazolamide, lipid component, and emulsifier were dissolved at 70 °C in ethanol to obtain an oily phase. The aqueous phase with Tween 80 and PEG 400 as surfactant and co-surfactants, respectively, was heated to the same temperature. After the addition of the oily phase into the aqueous one and removal of organic solvent, pre-emulsion was poured into the cold continuous phase and after 2 h of stirring, SLNs were obtained. Coated SLNs achieved a sustained drug release and reduced IOP for a longer time compared with both uncoated SLNs and commercially available formulation. The coated SLNs did not show any irritation. Another way for increasing drug penetration and absorption is to use cationic lipids when formulating SLNs. In vivo results obtained with this formulation demonstrated that it is possible to reduce the number of applications per day and improve patients’ adherence compared with the conventional eye drops. Nanoparticles showed a sustained drug release. After 1 h, 77.34% of methazolamide was released from the SLNs. There was a burst release because of either the dispersion of free drug in the external phase or the encapsulation of drug concentrated in the outer shell of the SLNs or on the nanoparticle surface. The large specific surface increased the initial drug release. The presence of phospholipids and surfactants provided an even larger specific surface area and smaller nanoparticle size. Thus, SLNs had a large external surface area, which contributed to a fast drug release. The AUC was 186.11–196.48 after 8 h, MRT was 5.16–5.28 h, and the maximum IOP reduction was 35.69–36.66 mmHg [140].

Leonardi et al. created cationic SLNs, size of 150–300 nm, of melatonin to treat glaucoma. Cationic lipid was used to increase the electrostatic interaction to negatively charged mucin. This interaction increased SLNs mucoadhessivness that led to enhancing drug penetration and absorption. In vivo data showed IOP reduction in albino rabbits of 7 mmHg at 6 h and 8 h, which lasted for 24 h. In vitro drug release study showed a sustained drug release that reached a plateau (35–60%) after 8–10 h. There were no signs of ocular irritation [141]

Surface-modified SLNs containing timolol with phospholipids, prepared by emulsification with high-pressure homogenization, can be an effective way to improve the ocular BA of timolol hydrogen maleate. In vitro drug release indicated a sustained drug release, because of the structure formed at the surface, which ensured drug release by diffusion, gradual erosion, or leaching [142]. Timolol-loaded CS nanoparticles, size of 143.9 ± 6.3 nm and zeta potential of 34.0 ± 6.4 mV, were more effective in IOP reduction compared with the timolol solution in rabbits’ eyes. The maximum IOP reduction was showed at 4 h (−8.4 ± 1.8 mmHg) and lasted for up to 8 h (−7.2 ± 0.3 mmHg). In vitro release studies showed that CS nanoparticles can effectively control drug release. Initial burst release (45–50% in 1 h, because of the drug fraction located at the nanoparticle surface) was followed by a slow drug release [127]. Timolol-propoxylated glyceryl triacrylate (PGT) nanoparticles, size of 3.5 nm, provided sustained drug release for up to one month in vitro. Timolol-PGT nanoparticles released timolol for an extended time because of the slow hydrolysis of the ester bond [143]. Zhao et al. [144] used galactosylated chitosan (GC) to develop timolol maleate-loaded nanoparticles, size of 213.3 ± 6.83 nm. This polymer is water-soluble at neutral pH, have better mucoadhesion and cell compatibility compared with the CS. In vitro study of drug release presented a sustained release compared with the eye drops: initial burst release of 36% in 1h (drug adsorption on the surface or weak encapsulation into the polymer) was followed by an extended-release of 90% in 8 h (the interaction between the encapsulated drug and the polymer and an increased mucoadhesion of GC that interacts with negatively charged mucin). The IOP-reducing effect had a maximum of 10.5 ± 0.51 mmHg after 4 h [144].

Acetazolamide-loaded Eudragit^®^ nanoparticles, size of 367 ± 8 nm and zeta potential of 7 ± 1.3 mV, displayed better permeability through the corneal tissue compared with the acetazolamide suspension, as well as a significant decrease in IOP and improvement of ocular tolerability. Acetazolamide-Eudragit^®^ nanoparticles were prepared by the solvent diffusion nanoprecipitation technique. Initial burst release during 2 h (because of both free drug and surface-adsorbed drug) was followed by a sustained drug release (a diffusive drug release from the Eudragit^®^ matrix) [145]. Transcorneal permeation study showed higher permeation of acetazolamide after 8 h with nanoparticles, size of 103.23 nm and zeta potential of −29.70 to −45.75 mV (74.50 ± 2.20 mg/cm^2^) compared with the eye drops (20.08 ± 3.12 mg/cm^2^) and suspension (16.03 ± 2.14 mg/cm^2^), whereas nanoparticles did not show any side effects on the corneal surface. Nanoparticles containing 1% poly(d, l-lactide-co-glycolide) (PLGA) reduced IOP for up to 8 h compared with the eye drops that reduced IOP for up to 2 h in rabbits’ eyes. Nanoparticles displayed biphasic release profile: initial burst release occurred because of the crystalline drug structure which was precipitated to amorphous structure in the PLGA matrix and shorter diffusion path length that increased in time, resulting in sustained drug release. The extended ocular hypotensive effect produced by NP-ISG can be attributed to the fact that the small size of ACZ-loaded PLGA nanoparticles facilitated higher binding to the corneal surface. The corneal surface became saturated with nanoparticles, thus sustained IOP reduction lasted up to 8 h because of the better transcorneal drug penetration into the anterior chamber [146].

#### 3.2.2. Nanoemulsions

Nanoemulsions (Figure 3B) are defined as nano-sized emulsions, manufactured for the improvement of drug delivery. They are thermodynamically stable isotropic systems in which two immiscible liquids are mixed to form a single-phase, using an emulsifying agent [147]. Acetazolamide-filled cationic nanoemulsions are more suitable in the treatment of glaucoma than anionic or neutral nanoemulsions because they reduced the production of aqueous humor more and thus lowered IOP to a greater extent. The range of droplet size was 240–443 nm. Anionic nanoemulsions showed a negative zeta potential of −36.9 mV, cationic nanoemulsions exhibited a positive zeta potential of 41.4 mV, whereas neutral-charged nanoemulsion had none. Drug release from cationic nanoemulsions was delayed compared with anionic and neutral-charged nanoemulsions. An increase of the dilution ratio from 1:5 to 1:40 did not show any sudden rise in the initial fraction released from any of the emulsions at 5 min. After 5 min, a progressive rise in drug release was observed. Cationic nanoemulsions showed a delayed drug release pattern in comparison with the other two nanoemulsions, which could be related to its ability to hold acetazolamide in the presence of competing anions, as determined in an in vitro study only [148]. Dorzolamide hydrochloride-filled nanoemulsions showed stable physicochemical properties and did not have irritable properties. The range of droplet size was 8.4–12.7 nm. The increase in oil content was not followed by an increase in droplet size. Increased number of oil droplets and reduced surfactant concentration led to an increase in the surface area of the globules and a thermodynamic activity of the drug, which acted as a driving force for a drug release. The maximum IOP reduction occurred after 0.5–1.6 h, and the effect lasted for up to 4–6 h (21.63–37.23%). The AUC was 85.49–130.53 after 10 h, and MRT was 2.01–2.86 h, as determined in an in vivo study in rabbits. They also demonstrated a fast onset of drug action and prolonged effect which led to an increase in BA. This formulation can decrease the number of daily applications and provide better patients’ adherence. Non-irritation and non-inflammation were shown, which is also very promising [149].

#### 3.2.3. Nanosuspensions

Nanosuspensions are defined as colloidal drug delivery systems with dispersed solid particles in the liquid phase. They can be easily integrated with hydrogels, because of their non-water-soluble characteristics. They are used for the delivery of lipophilic drugs, enhancing their BA [150]. Nanosuspension of coleonol reduced IOP by 31%, with drug effect lasting up to 12 h, which was significantly longer compared with the conventional formulations, as determined only by in vitro study [151]. Nanosuspensions of diclofenac, size of 105 nm, and zeta potential of 8 mV, increased drug retention time and its penetration in corneal tissues. The initial burst release (47% at 30 min) was followed by a sustained drug release (73% at 8 h). The AUC was 3.06 ± 0.57 µgh/mL, with a maximum concentration (Cmax) of 0.78 ± 0.11 µg/mL and tmax of 2 h, as determined by an in vivo study in rabbits. No ocular damage was observed in the cornea, conjunctiva, or iris 24 h post-administration, suggesting that this formulation has potential as an ocular drug delivery system in the treatment of glaucoma [152].

Ion exchange resins (IERs) are polymers with acidic groups (carboxylic and sulfonic) as cation exchangers, or basic groups (quaternary ammonium) as anion exchangers, which are involved in shielding and competitive binding to protect drugs. Betaxolol-loaded nanosuspension with IERs has been approved and commercially available. The cationic exchange resin containing 0.25% betaxolol increased the drug residence time in the cul-de-sac [153].

In a study that included patients with either primary open-angle glaucoma or ocular hypertension, no significant difference was found between 0.5% betaxolol solution and 0.25% betaxolol nanosuspension in terms of IOP reduction (3.6 mmHg and 3.3 mmHg, respectively), whereas ocular discomfort was significantly reduced for nanosuspension [154]. Nanosuspensions could be used as drug delivery systems for lipophilic antiglaucoma drugs, such as CAIs [155].

#### 3.2.4. Liposomes

Liposomes (Figure 3B) are defined as bilayers of phospholipids that are biocompatible with the human body and can deliver both hydrophilic and hydrophobic drugs [156,157]. They can improve drug BA and provide controlled drug release because they are responsive to certain triggers, such as temperature, electromagnetic waves, and pH value [158,159]. They can also degrade over time, based on the shell width, composition, and particle size [160].

The surface charge of liposomes impacts a drug residence time. The corneal epithelial surface is negatively charged because of the existence of a mucinous membrane, therefore positively charged liposomes bind easily, achieve prolonged residence time, and show greater corneal permeation and encapsulation efficacy compared with the negatively charged liposomes. Apart from positively charged liposomes, neutral liposomes also showed better properties compared with the negatively charged ones [161].

Positively charged timolol-loaded liposome formulations, size of 136.00 ± 18.00 nm and zeta potential of 2.75 ± 2.74 mV, reduced IOP to 11.96 ± 0.74 mmHg 1 h and 13.61 ± 0.95 mmHg 2 h after administration when incorporated into eye drops in rabbits’ eyes. Liposomes in deacetylated gellan gum (DGG) had a longer release time compared with liposomes on their own because DGG can form a gel after being affected by cations from tears, which can prolong drug release [162]. The higher concentration of cholesterol in lipid ratio led to forming larger dorzolamide-loaded liposomes, size of 5.68–76.14 nm. A burst drug release was noted at 1 h (70%) and was followed by nearly complete release (over 95%) at 6 h. The maximum IOP reduction was 4.42 mmHg at 4 h. The surface had a positive charge because of the cationic nature of dorzolamide, leading to the adhesion of nanoliposomes to the polyanionic corneal and conjunctival surface and improvement in corneal drug retention. Dorzolamide-loaded liposomes, size of 51 ± 3.24 nm, reduced IOP better (23.26 ± 9.24%) compared with the commercially available formulation (17.48 ± 7.62%) in rabbits’ eyes. The positive surface charge of liposomes helped their adhesion to the negatively charged cornea, as well as the similarity of the phospholipidic liposomal bilayer to the biological membrane and the small particle size [163,164].

After release from liposomes, drugs cross the corneal membrane through passive diffusion, so the longer the residence time, the higher the drug BA. For example, positively charged acetazolamide-loaded liposomes (size of 9.34 µm) showed bigger IOP reduction (−7.80 ± 1.04 mmHg) compared with the negatively charged ones (size of 7.94 µm) (−3.70 ± 2.18 mmHg) in rabbits’ eyes 3 h after administration, and this effect lasted longer (8 h vs. 3 h, respectively), as determined in an in vivo study in rabbits. Neutral multilamellar liposomes (size of 6.97 µm) showed promising results as they reduced IOP to 5.5 mmHg after 3 h, and the effect lasted for 8 h. The increase in cholesterol molar ratio decreased a drug release from liposomes. Acetazolamide was embedded in the hydrophobic regions of liposomes, thus its release would occur over a prolonged time. No signs of irritation or inflammation were noted. Thus, this is a promising ocular drug delivery system in the treatment of glaucoma [165].

Monem et al. studied the effect of the surface charge of liposomes on IOP reduction in rabbits. Neutral liposomes loaded with pilocarpine provided a similar IOP reduction in rabbits’ eyes, but it lasted two times longer, thus increasing a drug residence time by 10 h and reducing dosing frequency, compared with the conventional eye drops or negatively charged liposomes. For neutral liposomes, the IOP reduction was from 20.7 mmHg to 15 mmHg after 30 min and the reduced IOP remained for 4–5 h. Negatively charged liposomes showed shorter (nearly one third) drug action compared with the neutral liposomes [158].

Latanoprost-loaded egg-phosphatidylcholine liposomes, size of 109 ± 18 nm administered subconjunctivally enhanced stability and reduced IOP for up to 90 days in rabbits’ eyes (4.8 ± 1.5 mmHg), compared with the topical administration of latanoprost [160,166].

The disadvantage of liposomes could be their tendency to aggregate, which can cause a drug leakage. They are also prone to the process of phagocytosis, but the surface-modification has helped overcome these problems. Bioadhesive polymers are used to coat liposomes, which inhibits their aggregation and increases their viscosity [167].

Polymers used for coating are usually either poly-L-lysine or CS [168,169]. For example, timolol-loaded CS-coated liposomes, size of 150.7 ± 3.82 nm and zeta potential of 16.1 ± 0.59 mV, reduced IOP better (19.67 ± 1.14 mmHg) compared with the timolol eye drops (23.80 ± 1.49 mmHg) in rabbits’ eyes. In vitro, the initial burst was 29.05% ± 1.33 in 1 h. Almost complete release (78.59 ± 1.68%) was noted in 10 h and 12 h. This showed that liposomes exhibited sustained drug release [170]. Bubble liposomes are a promising strategy for gene delivery through ultrasonication [171]. When coenzyme-Q10 was incorporated into liposomes of 100–200 nm, a great anti-cataract effect, superior superoxide dismutase activity, and glutathione reduction were achieved [172], whereas the same formulation with bevacizumab could pass through barriers via annexin-A5-mediated endocytosis [173].

Latanoprost-loaded liposomes, size of 103.18 ± 5.1 nm, were administered subconjunctivally to six humans who were diagnosed with either ocular hypertension or primary open-angle glaucoma. The injection was well tolerated by all six of them. Baseline IOP was 27.55 ± 3.25 mmHg, whereas a mean IOP decrease was 13.03 ± 2.88 mmHg or 47.43 ± 10.05%. A clinically and statistically significant IOP reduction (≥20%, *p* < 0.05) was observed after three months. Sustained IOP-lowering effect was caused by both retention of latanoprost within the anterior chamber and a sustained drug release from the liposomes, but it could also be that the bound drug was not cleared away fast from the eye [174].

#### 3.2.5. Niosomes

Niosomes (Figure 3B) are defined as non-toxic spherical closed bilayer structures of non-ionic amphiphiles (surfactants) that can deliver both hydrophilic and hydrophobic drugs simultaneously, which could be useful in combined drug therapy. They provide enhanced drug BA and positive therapeutic responses [175,176].

Timolol-loaded CS-coated niosomes, size of 2–3 µm, exhibited 1.7 and 2.34 times higher Cmax and AUC, respectively, in aqueous humor compared with the timolol solution. Timolol was released slowly from niosomes and stayed in aqueous humor for a longer time, thus an effective inhibition of aqueous humor production was achieved, with a subsequent increase in the concentration of timolol (secondary peak), because the volume of aqueous humor decreased considerably. Timolol in a solution was washed off quickly, exhibiting a significantly smaller secondary peak. In rabbits’ eyes, timolol-loaded niosomes coated with CS provided longer IOP reduction (8 h) compared with the conventional formulations (2 h), and Carbopol-coated niosomes (6 h). The use of niosomes provided fewer systemic side effects [177,178]. Acetazolamide-loaded niosomes also showed prolonged drug release in the eye. With niosomes, there was a maximum IOP reduction of 3 mmHg (7% more than the acetazolamide suspension) and the effect was maintained for up to 5 h. In the case of bioadhesive-coated niosomes, the maximum effect was maintained for up to 6 h. The coated niosomes provided 33% IOP reduction, whereas the uncoated niosomes provided 30% IOP reduction. The Cmax was 14.94 µg/mL, tmax 100 min, and AUC 1230.0116 µgmin/mL, for the bioadhesive-coated formulation [179]. Multilamellar niosomes can entrap a higher drug dose and release it for a longer time [180].

#### 3.2.6. Dendrimers

Dendrimers (Figure 3B) are defined as biocompatible and non-immunogenic polymeric materials with flexible branching and large surface area [181,182].

Hybrid poly(amidoamine) (PAMAM)-dendrimer hydrogel-PLGA nanoparticles with an acrylate-carrying PEG for the codelivery of two traditional antiglaucoma drugs, brimonidine and timolol, showed the absence of cytotoxic effects, as well as prolonged IOP reduction and retention time with delayed drug release, thus improving their ocular BA in aqueous humor for seven days in rabbit’ eyes, compared with the saline control. Both brimonidine and timolol were slowly released in vitro for 28–35 days. Particles had a size of 258 nm and a zeta potential of −28.8 mV. In vitro and in vivo studies showed that dendrimers provided sustained release of brimonidine and timolol, as well as an effective IOP reduction (18%) for four days. This way the dosing frequency could be reduced, which could significantly improve patients’ compliance. Responses from patients are expected to be positive [115,183]. Also, PAMAM dendrimers with amine and carboxylic terminal ends showed a longer residual time of pilocarpine (up to 5 h), significantly reduced ocular irritation, and provided greater drug BA, compared with the commercially available eye drops. Lacrimal drainage washed out some of the dendrimers, but they still exhibited improved mucoadhesiveness. The pharmacodynamic study emphasized greater drug BA when dendrimers with carboxylate and hydroxyl surface groups were combined with the eye drops. The information about the influence of the physicochemical properties of polymeric macromolecules on the drug residence time will improve the design of novel polymeric ophthalmic systems with prolonged drug release pattern [184].

Holden et al. created a gel consisting of PAMAM dendrimers with PEG-acrylate chains loaded with brimonidine or timolol. When exposed to light, PEG-acrylate chains cross-link and form a solution. This gel is non-toxic and showed mucoadhesiveness, as well as increased drug BA in the epithelium, stroma, and endothelium. In vitro, dendrimers showed a sustained drug release: 72 h for brimonidine and 56 h for timolol, because of the drug entrapment in the PEG network and the drug encapsulation. Drugs in eye drops were released more quickly, within 1.5 h, indicating that the eye drops did not exhibit a sustained drug release [185].

Carteolol-loaded dendrimers with a carboxylic terminal group and quaternary ammonium salt showed longer residence time and no ocular irritation in rabbits’ eyes, which can reduce dosing frequency. Drug BA in aqueous humor was 2.5 times higher compared with the conventional solution [182]. Acetazolamide-loaded carbosilane dendrimers were well tolerated at a concentration of 10 μM and showed an IOP reduction of 22.6% in rabbits’ eyes, which was significantly higher compared with the acetazolamide solution (17.2%). The AUC after 8 h was 1.25 times higher for dendrimers compared with the acetazolamide solution. The efficacy was therefore significantly higher for dendrimers than solution [186]. Dendrimers of 50 nm provided effective gene transfection in RPE cells [187].

#### 3.2.7. Cyclodextrin Complexes

Cyclodextrins are defined as cyclic oligosaccharides with a ring-like structure consisting of sugar molecules. They can be classified as α-, β-, and γ-cyclodextrins containing 6-, 7-, and 8-membered rings, respectively [188]. Their advantage is that they can deliver a drug without changing its molecular structure. Cyclodextrins have a hydrophilic surface, so an entrapped lipophilic drug can pass through the hydrophilic barrier of the eye and reach the lipophilic corneal surface, to be released in aqueous humor [67].

Dorzolamide with γ-cyclodextrin showed prolonged residence time in aqueous humor and could be used as a once-daily formulation. The complex had a size of 5.4 µm. The Cmax was 5.4 µg/mL and tmax of 4 h. Sustained release of dorzolamide was shown, as well as sustained high dorzolamide concentrations in aqueous humor for up to 24 h. It can be concluded that the complex of dorzolamide and γ-cyclodextrin has the potential to be one of the best formulations for ocular delivery of that drug [189].

The sulfobutyl ether of the β-cyclodextrin complex showed a protective effect on pilocarpine before it reached the target area. It reduced the ocular irritation caused by this drug by preventing its rapid absorption and precipitation in the pre-corneal area [190,191]. The complex of hydroxypropyl β-cyclodextrin and triethanamine (TEA) with acetazolamide can release this drug for up to 4 h and cause IOP reduction by 30%. Higher solubility of acetazolamide was presented when it was combined with cyclodextrins, which could result in more effective corneal drug delivery. An IOP reduction of 30% was achieved with the ternary system (with TEA) for 4 h, and 6% for 3 h with the binary system (without TEA). The addition of TEA could prevent the complexation of acetazolamide with hydroxypropyl β-cyclodextrin, thus a larger amount of free drug could be available for the ocular absorption [192]. Brinzolamide-loaded hydropropyl β-cyclodextrin systems, size of 82.29 ± 6.20 nm and zeta potential of −3.57 ± 0.46 mV, reduced IOP in 2 h by 32.3% and showed a sustained drug release effect for 12 h, compared with the brinzolamide suspension. In vitro release study showed moderate sustained release of brinzolamide within a period of 9 h. An enhanced IOP reduction was also presented when compared with the brinzolamide suspension: the maximum IOP reduction was 32.3% at 2 h and lasted for 12 h. Even though the dosage of brinzolamide was just 10% in the cyclodextrin system compared with the suspension, the IOP reduction was greater, as determined in an in vivo study in rabbits. This formulation showed similar safety as the commercially available formulation, proving its great potential in the treatment of glaucoma [193]. Propylamino-β-cyclodextrin showed a significant improvement in solubility and stability of latanoprost in vitro and 6% less ocular irritation and inflammatory mixed cell infiltrates in vivo, compared with the commercially available formulation. The complexation of latanoprost with propylamino-β-cyclodextrin increased the solubility of latanoprost thus avoided a potential drug loss related to its adsorption on the surface. This formulation decreased ocular irritation compared with the commercially available latanoprost formulations, as well as improved drug delivery by increasing the retention time of latanoprost, as determined in an in vivo study in rabbits. Latanoprost with propylamino-β-cyclodextrin showed better ocular tolerance compared with the commercially available formulation. This is a promising solution for the long-term treatment of glaucoma, especially because of the improved patients’ adherence [194].

#### 3.2.8. Nanocrystals

Nanocrystals are defined as solid particles with a diameter of less than 1 μm and a crystalline structure. They are composed of a drug and a crystalline coating [195]. They can provide an effective surface area and show a good drug BA without carriers. Nanocrystals provide fast initial dissolution, indicating better drug BA, but after 1 h, all nanocrystals dissolve, which is compatible with the commercial formulation [196].

Nanocrystals of brinzolamide, size of 460 ± 10 nm and 530 ± 2 nm, showed better IOP reduction (75%) in rats’ eyes compared with the commercially available formulation (49%). The HPMC was an effective stabilizer for maintaining the reduced particle size and preventing aggregation. In contrast to the polymeric nanoparticles, nanocrystals have clear regulatory guidelines, since they have no matrix and only consist of a drug and a stabilizer [197]. Pilocarpine-loaded cellulose nanocrystals with triblockpoloxamer copolymer showed greater sustained drug release and less toxicity. In vitro, a sustained release of pilocarpine from the nanocrystals was achieved within 20 h compared with the pure poloxamer gel. At 7 h, the maximum drug release was 87.26% [198]. Trimethyl lock brinzolamide nanocrystals showed similar efficacy compared with the commercially available formulation at one-fifth of a molar concentration without toxic effects on the cornea in rats’ eyes. The hydrolytic behavior of brinzolamide nanocrystals was related to a greater IOP reduction [199].

#### 3.2.9. Nanodiamonds

Nanodiamonds (Figure 3B) are defined as carbon nanoparticles with a truncated octahedral structure with sizes of 2–10 nm [200]. Many functional groups on their surface are covalently or non-covalently conjugated. For example, their surface coated with polyethyleneimine (PEI) can be conjugated with *N*-acetylated CS and loaded with timolol to prepare a nanogel in the form of contact lenses (CLs). Therefore, sustained drug release can be achieved as CS dissociates in the presence of lysozyme. Nanodiamonds also provide the mechanical support of CLs [201].

### 3.3. Ocular Inserts

One of the most important challenges in the formulation of ocular inserts is their shape as it affects drug loading capacity, retention, and comfort. It is not been yet clear which shape of the ocular insert is most functional, but it has been shown in human volunteers that rod-shaped devices are better tolerated [202].

The first reported use of any type of ocular inserts was a small portion of filter paper impregnated with a drug solution, e.g., atropine sulfate or pilocarpine hydrochloride [203]. Ocular inserts (Figure 3C) are generally classified as insoluble or soluble, although some authors introduced the term bioerodible as a third type. Although some consider soluble and bioerodible inserts to be the same, they differ essentially because of different underlying chemical processes [204]. This classification is based on the polymers used in the formulation of ocular inserts. The most commonly used polymers in these formulations are MC and its derivatives, HPMC, ethylcellulose (EC), polyvinyl pyrrolidone (PVP), polyvinyl alcohol (PVA), CS and its derivatives, gelatin, and different mixtures of these polymers [205]. To date, most devices for posterior drug delivery, such as IOP-lowering drugs for glaucoma treatment, have been researched) [202].

An example of an insoluble ocular insert is Ocusert^®^ (Alza Corporation Inc., Vacaville, CA, USA) developed by Armaly and Rao (Figure 3C). Pilocarpine is incorporated into a core of alginate gel, which is a central reservoir surrounded by a microporous membrane of ethylene-vinyl acetate copolymer. Pilocarpine diffuses in a controlled manner through this membrane [206,207]. Ocusert^®^ is withdrawn from the market because of burst drug release and dislocation problems [202].

Soluble Ophthalmic Drug Inserts (SODI) are small oval plates made of polyacrylamide, vinyl pyrrolidone, and ethyl acrylate copolymers developed by Maichuk. After administration in the cul-de-sac, SODI soften and turn into a viscous polymer solution after 60–90 min upon contact with the tear film. A drug is released throughout 34–72 h [208]. It is withdrawn from the market for unknown reasons [202].

Reservoir-type ocular inserts of brimonidine tartarate were successfully formulated by the solvent casting technique, using 1.41% cellulose acetate butyrate (CAB) as a rate-controlling membrane and 5% HPMC as a reservoir film. The PEG-600 was used in the formulation as a plasticizer, at a concentration of 60% of the dry weight of CAB. The formulation showed a controlled drug release throughout 24 h with an excellent in vivo/in vitro correlation, as the IOP-lowering effect also lasted 24 h. Inserts had an acceptable thickness, which meant they were non-irritable to the eye, as proved in the eye irritancy test, and also showed good stability and physical integrity [209].

Biodegradable ocular inserts, obtained by solvent casting technique from 7% PVP K-90, 1.5% low molecular weight SA with unilateral EC coating, were able to sustain the in vitro release of brimonidine tartarate for 24 h. Their therapeutic efficacy in terms of IOP reduction was superior to conventional brimonidine tartarate eye drops. However, the IOP decrease lasted seven hours, which was not in line with the in vitro findings, but still represents a sustained release. When applied, this formulation caused mild lacrimation without redness. This may be a promising approach, as the authors say, but one question remains: Would it be practical for the patient to see a doctor every 7 h to get another insertion [210].

Mealy et al. developed a brimonidine tartarate-releasing ocular insert designed from PLGA/PEG. The formulation containing 15% PEG released brimonidine tartarate linearly for 30 days. Besides, these inserts had smooth surfaces and physical properties suitable for ophthalmic use. The results of the Chang conjunctival epithelial cell test showed that the formulation was minimally toxic, but not significantly different from the control group. This study did not provide in vivo IOP lowering results so IVIVC remains unknown [211].

Ocular inserts with CS alone are being developed for various antiglaucoma drugs, such as bimatoprost [212], brimonidine tartarate [213], diminazene [214], in a combination with hydroxyethyl cellulose for dorzolamide [215] or in a combination of equal parts with chondroitin sulfate for diminazene [216].

#### 3.3.1. Ocufit SR^®^ System

The Ocufit SR^®^ system (Figure 3C) is a drug-eluting, rod-shaped ocular device that can be inserted into the lower and upper conjunctival fornix [202]. The cylindrical rod, shaped and dimensioned to fit into the human fornix, was manufactured from a silicone elastomer and loaded with timolol. The study showed that the positioning of this system in the upper conjunctival sac leads to increased ocular drug absorption [204].

#### 3.3.2. Topical Ophthalmic Drug Delivery Device

The Topical Ophthalmic Drug Delivery Device (TODDD™), developed by Amorphex Therapeutics (Andover, MA, USA), is a soft, flexible, topical ocular device made of clear elastomeric material, worn under the upper eyelid in contact with the conjunctiva (Figure 3C). Pre-clinical trials of TODDD™ containing timolol, prostaglandins, or their combination have been reported, which can deliver multiple medications and ensure continuous delivery for up to 90 days. Leahy et al. evaluated the efficacy of TODDD™ with timolol in normotensive rabbits. They found that a maximum IOP reduction was maintained throughout the entire three-month trial period [217]. Another pre-clinical study in eight Beagle dogs using TODDD™ with latanoprost showed IOP reduction comparable to that of timolol. However, retention rates were low and at the end of the study period of 16 days, only three devices were in place, which could however be due to the third eyelid (nictitating) membrane of the animals. It cannot be said with certainty that the same would be the case in humans. Both studies showed no systemic drug concentrations [218].

#### 3.3.3. Topical Ocular Ring

An interesting type of ocular inserts is the topical ocular ring (Figure 3C). It is a preservative-free formulation containing 13 mg of bimatoprost blended into a silicone matrix, which is placed over an inner polypropylene carrier structure and produced in diameters ranging from 24 to 29 mm. The release rate of bimatoprost into the tear film is determined by the physical properties of the silicone, the surface area of the silicone-drug matrix, and a concentration of bimatoprost in the silicone-drug matrix. For six months, bimatoprost inserts released a decreasing drug dose, from approximately 35 mg daily (on day 0) to 6 mg (on day 180). Formulation tests have been conducted to examine if travoprost or latanoprost could also be incorporated into the silicone matrix. As the ring has a relatively large volume for a sustained-release system, it can administer two drugs, e.g., bimatoprost and timolol. Also, clinical trials have confirmed that IOP reduction, when applying this ring, lasts for six months. It has been proven to be safe and well-tolerated. It comes in various sizes so patients can choose the right one, and has excellent primary retention rates [219,220].

#### 3.3.4. Punctal Plugs

Punctal plugs (PPs) block the tear drainage and prevent natural tears from exuding on the eye surface (Figure 3C). In the last ten years, several solid or semi-solid models have been developed with additional drug-releasing functionality that can be used in glaucoma treatment for sustained drug release for up to four months. The basic idea was that when the tear drainage is blocked, locally administered drops are prevented from leaving the eye quickly, thereby prolonging a drug residence time and drug penetration and thus improving its effectiveness. Simple insertion of PPs makes them widely accepted as drug delivery systems. In some patients, however, foreign body sensation after their application can be a significant obstacle. The PPs can be used either as aids that block the tear drainage or as drug delivery systems. Depending on the material used, PPs can be classified as semi-permanent or temporary. Silicone, Teflon^®^, hydroxyethyl methacrylate (HEMA), polycaprolactone (PCL), or polydioxanone are most commonly used for semi-permanent PPs, while animal collagen and various polymers are used for temporary PPs. Semi-permanent PPs are removed spontaneously or by a doctor. On the other hand, all temporary PPs are removed spontaneously by dissolving within a period of three days to six months, depending on the material they consist of [221]. The biggest disadvantage of PPs is that due to their size very limited quantities of drugs can be loaded [202].

The PPs efficacy as aids was confirmed by Opitz et al., who demonstrated the additional IOP-lowering effect when travoprost monotherapy was combined with PPs [222]. Sherwin et al. came to similar results in patients taking various IOP-lowering agents, including fixed-dose combinations of PAs [223]. When it comes to PPs as drug delivery systems, a drug can be loaded in two ways. It can be in the core, inside an impermeable layer from which it diffuses through the cross-section. The second method involves coating the plugs with a drug solution [221].

Travoprost PP developed by Ocular Therapeutix Inc. (Bedford, MA, USA) is a rod-shaped PP consisting of poly (lactic acid) (PLA) microspheres with encapsulated travoprost embedded in a dried PEG-hydrogel matrix. Bioresorbable microspheres are slowly degraded by hydrolysis in the tear fluid and release this drug for over 90 days. This system is equipped with a visual aid to support post-placement visualization [224,225]. Mati Therapeutics developed L-shaped latanoprost PP, with latanoprost embedded in a polymer mixture (Evolute^®^ platform) in a reservoir. After contact with the tear film, this drug is released through an orifice for three months [221,226].

Envisia Therapeutics Inc. (Morrisville, NC, USA) developed travoprost PP (ENV515) using PRINT^®^ technology to adapt to the anatomy of the human iridocorneal angle [227]. The ENV515 is a drug delivery system based on biodegradable nanotechnology polymers, while PRINT^®^ technology (Liquidia Corporation, Morrisville, NC, USA) is a particle-engineering platform enabling particle replication in non-wetting template technology [225]. The ENV515 demonstrates in vitro sustained release of travoprost and in vivo IOP-lowering effects for six months [227].

Jacob et al. believed that the obstacles to the formulation of antimitotics could be solved by focusing drug release on the site where the tissue thickens thereby increasing the life span of the bleb. In their study, they chose 5-FU because of the lower toxicity compared to MMC, because they expected prolonged exposure to the drug. They placed collagen plugs containing 5-FU into the silicone tubes of the modified Baerveldt glaucoma drains and implanted these and placebo glaucoma drains into the right eye New Zealand rabbits for in vivo evaluation. They observed that eyes that received 5-FU plugs had a physiologically significant decrease in IOP by the end of the first week. This effect remained evident during the first three postoperative months but disappeared six months after implantation [228].

### 3.4. Contact Lenses

The possibility of using CLs (Figure 3D) as drug delivery systems has also been the subject of numerous studies. Some of them have shown that soft CLs provide sustained drug release but must remain transparent in order not to impair vision, which is the biggest challenge in their formulation. Soft CLs are a network formed after the cross-linking of water-soluble polymeric hydrogels. Numerous polymers have been used in their formulations [202,207,226].

The first study of CLs for glaucoma therapy dates back to 1974 when Hillman soaked vinyl pyrrolidone/acrylic copolymer CLs in 1% pilocarpine eye drops for three days. Its IOP-lowering effect was equivalent to that of a 4% pilocarpine solution [229]. A newer approach is to encapsulate timolol microemulsion in poly-2-hydroxyethyl methacrylate (p-HEMA) hydrogel lens, which releases timolol for eight days [230]. To improve the control of drug release from the p-HEMA lens loaded with timolol microemulsion, Li et al. formulated a microemulsion of ethyl butyrate in water and stabilized it with PF-127 as a surfactant. This variant did not have the desired effect, as in vitro release profile in phosphate buffer saline showed a rapid release of timolol compared with that of deionized water [231].

An improved approach was to load CLs with vitamin E, as vitamin E has been shown to increase the timolol release time [232]. Peng et al. used ACUVUE^®^ TruEye™ lenses (Johnson & Johnson Vision Care, Inc., Jacksonville, FL, USA), which present an innovative design that provides comfort and maintains a constantly lubricated surface, resulting in a lens that clinically provides comfort comparable to no lens-wear at all [233]. They were soaked in a vitamin E-ethanol solution and then in a timolol maleate solution in the phosphate buffer. In vivo results showed that IOP can be significantly reduced by continuously wearing ACUVUE^®^ TruEye^TM^ (Johnson & Johnson Vision Care, Inc., Jacksonville, FL, USA) with 20% vitamin E. The IOP was lowered for five days, although the drug was not administered beyond the fourth day, which may be due to the accumulation and subsequent release of the drug in ocular tissue. No signs of discomfort or ocular toxicity were observed [234].

Hsu et al. used ACUVUE^®^ OASYS™ CLs (Johnson & Johnson Vision Care, Inc., Jacksonville, FL, USA) and loaded them with vitamin E as described in the previous study. Subsequently, CLs were loaded with a combination of timolol and dorzolamide. This combination was used as there are commercially available eye drops containing these two drugs. In vitro release profiles showed the 48-h prolonged release, with the same duration of IOP-lowering effect as recorded in in vivo studies in Beagle dogs [235].

Latanoprost-eluting CLs were developed by Ciolino et al. by encapsulating latanoprost-PLGA films in methafilcon lenses by ultraviolet light polymerization [236]. Methafilcon lenses are copolymers of HEMA and methacrylic acid [237]. In vitro and in vivo studies have shown an early onset of drug release followed by a sustained drug release for one month [236]. When applied to cynomolgus monkeys, these CLs provided sustained delivery of latanoprost at least as effectively as latanoprost eye drops applied daily and possibly more [238]. Sun et al. loaded brimonidine onto layered double hydroxide (Bri@LDH) consisting of numerous micelles based on PLGA-PEG-PLGA copolymer. In vivo drug release from the special contact lens made of Bri@LDH/Thermogel was maintained for at least seven days, effectively modulating IOP relief [239].

Five commercially available silicone CLs including ACUVUE^®^ ADVANCE^TM^ and ACUVUE^®^ OASYS™ (Johnson & Johnson Vision Care, Inc., Jacksonville, FL, USA), NIGHT & DAY™ (Alcon, Fort Worth, TX, USA), O2OPTIX™ (Alcon, Fort Worth, TX, USA), and PureVision™ (Bausch & Lomb, Bridgewater, NJ, USA), were used in the study to increase the release time of dexamethasone by loading vitamin E into the CLs. In contrast to hydrophilic drugs, where vitamin E in CLs is a hydrophobic diffusion barrier, the diffusion of hydrophobic dexamethasone is reduced thanks to the viscosity of vitamin E. The vitamin E was loaded into the CLs by soaking the lenses in an ethanolic solution of vitamin E followed by ethanol evaporation. CLs with 30% vitamin E-laden extended the release of dexamethasone to 7–9 days, which was 9−16 times higher than CLs without vitamin E. Another important finding of this study is that vitamin E reduced the release of dexamethasone by more than 25% in the first 5 h [240].

It was proven that better control of dexamethasone release could be achieved by using hydrophobic CLs. In a study by Behl et al., CS NPs synthesized by ionic gelation were dexamethasone-laden and enclosed in hydroxy methacrylate-ethylene glycol dimethacrylate CLs. CLs loaded with 200 μg NPs maintained 95% optical clarity and showed a continuously increasing dexamethasone release for 10 days with the total amount being released over 22 days and an increase in BA of 72% compared to conventional eye drops [241]. Additional studies on cytotoxicity and in vivo effects are still pending, but this formulation solved the problems of sustaining dexamethasone release and is convenient for patients to improve treatment adherence.

### 3.5. Collagen Corneal Shields

Collagen corneal shields (CCSs) are inserts similar to CLs, composed of collagen of bovine or porcine origin. They are impregnated with a drug by immersing in its solution. They have been introduced for the treatment of post-traumatic conditions or corneal protection. They are not practical for the treatment of chronic diseases, because they develop their effect over several days. This would mean frequent lens replacement in chronic diseases, such as glaucoma, which is not practical for a patient or a physician. After application to the eye, CCSs dissolve and form a viscous protective layer that covers the corneal surface and thus protects it. The collagen matrix acts as a reservoir, to which a drug is reversibly bound. A drug is gradually released and thus the occurrence of systemic toxicity is prevented. They are produced in a dehydrated form and must be moisturized before application [242,243].

Agban et al. developed a novel PVP-capped zinc oxide (ZnO/PVP) collagen shield cross-linked with pilocarpine hydrochloride. The ZnO/PVP showed a transparency rate of 79.3% compared with water and a continuous pilocarpine hydrochloride release for over 14 days [244].

### 3.6. Ocular Implants

Ocular implants (Figure 3E) as drug delivery systems can be made of polymers, stainless steel, or other metals and can be biodegradable or non-biodegradable. Biodegradable implants can be made of PLGA, silicon, or PLA. They degrade after a certain time in the eye and are absorbed. In contrast to them, non-degradable implants need to be surgically removed and this removal could cause some complications. However, their advantage is a longer drug release. They can be made of metals or polymers such as PVA and ethylene-vinyl alcohol [245]. Kim et al. developed an implant with a PA called DE-117. This drug is encapsulated between two PCL films. The PCL is a biodegradable polymer that limits drug diffusion and allows an extended drug release of this formulation for six months [246].

Allergan plc (Dublin, Ireland) has commercially developed Bimatoprost SR, a biodegradable implant that releases bimatoprost in a sustained manner for over six months. It contains bimatoprost within the biodegradable NOVADUR^TM^ drug delivery system (Figure 3E). The NOVADUR^TM^ system is based on polyglactin PLGA, which is slightly modified to ensure a uniform release of bimatoprost for up to six months [247]. The company is also developing a brimonidine-NOVADUR^TM^ drug delivery system [248].

PolyActiva Pty Ltd. (Parkville, Australia) has developed a rod-shaped latanoprost ocular implant that delivers a constant daily dose of latanoprost for at least six months from the first day after administration. Designed to biodegrade as quickly as possible after the treatment, the implant is biodegraded into safe and non-toxic by-products without leaving any residues. The implant consists of a single biomaterial (latanoprost free acid polymeric prodrug) engineered from PolyActiva’s polytriazole hydrogel system. The safety of this implant was demonstrated in a phase Ia safety study and is currently being evaluated in a phase Ib clinical trial [249].

Samy et al. developed a PCL implant for codelivery of timolol and brimonidine. Two PCL thin-film pockets were loaded, one with timolol and another one with brimonidine, and connected to form a V-shaped codelivery device. In vitro release tests showed sustained release of both drugs for over 60 days. An in vivo study demonstrated IOP reduction for over 13 weeks [250]. The iDose is a titanium implant filled with a travoprost formulation and covered with a membrane designed to release this drug in a sustained manner into the anterior chamber. The iDose’s efficacy is compared with that of timolol solution in a randomized phase II-clinical trial, where it demonstrated its superiority over conventional solution. The iDose shows drug release and IOP reduction for 12 months, which is more than any other novel drug delivery system. However, its main drawback is that is non-biodegradable and must be surgically removed once the total drug amount has been released. This study also showed a favorable safety profile for the iDose, without side effects such as hyperemia [225,251].

Another interesting approach in coping with glaucoma is the use of micropump technology, commercially developed by Replenish, Inc. (Pasadena, CA, USA) under the brand name Ophthalmic MicroPump^TM^ System. They developed four subsystems: Anterior MicroPump™ for patients with glaucoma, Posterior MicroPump™ for patients with retinal conditions, EyeLink™, and Drug Refill System™. A pump is implanted directly into the eye. A drug is injected through a port into a reservoir and released via a cannula system. The amount of drug release and dosing frequency is controlled by EyeLink™, a wireless programmer/charger for bi-directional communication with MicroPump™ implants.

The Drug Refill System™ is a separate console unit used to fill and refill MicroPump™ implants with a drug. A disposable refill-tubing kit with the 31-gauge needle is used to fill and refill the implant [252].

One of the solutions to obstacles in formulating dexamethasone came in the form of the FDA and European Medicines Agency approved dexamethasone intravitreal implant called Ozurdex^®^ commercially developed by Allergan plc (Dublin, Ireland). This implant is intended for delivery in the anterior and posterior eye chamber and can be successfully used in glaucoma, as glaucoma is considered to be both an anterior and posterior ocular condition. Anterior, as the goal of therapy, is to reduce the IOP in the anterior chamber, and posterior, as the therapy seeks neuroprotection of the optic nerve [253].

This system is manufactured by double melt extrusion, resulting in bioerodible microspheres made from biodegradable PLGA copolymer and micronized dexamethasone. These implants are inserted into the eye through a small hollow gauge needle after making a small incision in the sclera. This system showed a sustained in vitro release during the testing period of 45 days [253], while it has been shown to be therapeutically effective over 6–9 months [254].

This system has proven to be effective, safe, and well-tolerated [255], with the limitation of delivering only minimal drug concentrations into the anterior chamber, and may have potential complications such as elevated IOP and retinal detachment [256] as well as keratitis as a result of herpes simplex virus reactivation after implant injection [257].

Bi et al. tried to solve the problem of scarring by fixing 5-FU-poly(ε-caprolactone) sustained-release film to the Ahmed glaucoma valve (AGV) implant with a 10-0 suture. Sustained-release films were prepared by spraying methods. These implants were tested in vitro and in vivo after the implantation of AGV in rabbit eyes. In vitro results showed sustained release over three months with a cumulative release of about 90%. According to in vivo results, they succeeded in keeping the drainage of the aqueous humor uninterrupted for three months, since the IOP, central depth of the anterior chamber, and bleb formation showed no significant changes [258].

AGV was also a substrate for film layering in the work of Ponnusamy et al. They designed a system containing 5-FU and/or MMC in PLGA film, which was then coated on AGV. They offered a very interesting solution in creating films since they used the “breath figure” method, a simple method which aimed at creating a regular arrangement of the pores in a polymer film, when the solvent evaporates under humid conditions, leaving a structure similar to honeycombs [259,260]. Their solution was indeed worth mentioning because they assumed that the porous structure would be beneficial both in controlling drug delivery and in the degradation of the polymer. To achieve continuous-release, they produced double-layered porous PLGA films in which 5-FU was dispersed into the bottom layer and MMC was applied to the top layer. Thus, the reduced dose of each antifibrotic could be used. The system was designed to provide a burst release of a small dose of MMC to limit the infiltration of immune cells immediately after surgery, and a slow release of less potent 5-FU over an extended period to inhibit fibroblast proliferation. At the beginning of the release of 5-FU, PLGA began to degrade. Results from the study showed that the inhibition of fibroblast growth continues 3–4 weeks after surgery during which most wound healing occurs [261].

### 3.7. Microneedles

Microneedles (MNs) represent a third-generation of minimally invasive devices, originally designed to enhance transdermal drug delivery for various active substances. Upon administration, they perforate the stratum corneum with limited interactions with pain receptors (nociceptors) located in the dermis [262,263,264]. These devices are composed of needle-like solid or hollow projections of micron dimensions (25–2000 µm) and fabricated of a wide range of materials including silicon, stainless steel, ceramic, glass, sugar, metal, and polymers [263,264,265].

Although MNs are extensively investigated for transdermal delivery of active substances, they have recently been proposed for targeted transscleral delivery of therapeutic entities into ocular tissues [262]. Previous studies have confirmed that MNs have successfully delivered active substances into both posterior and anterior segments of the eye. They have a suitable length to overcome ocular barriers such as the epithelial transport barrier and the conjunctival clearance mechanism, thus allowing intrascleral and intrastromal delivery while minimizing the potential for retinal damage [264,266]. Therefore, they provide a precise drug deposition within ocular tissues, and drugs can be transported to the site of action by diffusion (Figure 3F) [266].

For effective treatment of glaucoma, active agents should permeate through tear fluid, cornea, and conjunctiva [265], thereby reducing the risk and/or complications associated with intravitreal injections such as cataract, hemorrhage, (pseudo)endophthalmitis, and retinal detachment [267]. The design of MNs enables hygienic, safe, user-friendly, and minimally invasive applications without damaging deeper ocular tissues, as they penetrate only a few hundreds of micrometers into the sclera [117]. Thus, they release drugs into sclera or space between sclera and choroid that goes circumferentially around the eye, termed as suprachoroidal space (SCS) [267]. Although five different types of MNs are described in the literature, the ocular application is focused primarily on only three [264,265,268]:

- Coated MNs–that perforate the ocular tissue and then, the drug formulation is dissolved within minutes of insertion, followed shortly thereafter by device removal [266],
- Hollow MNs–that enable the effective administration of drugs within the ocular tissue through passive diffusion or pressure, from external reservoir [269,270,271,272], and
- Dissolving polymer MNs–composed of a soluble matrix that completely dissolves when administered [273].

These three types of MNs enable rapid drug delivery and retrieval of either the MNs or their baseplates, thus imitating the application of conventional hypodermic needles. Solid and hollow MNs can be used to administer a wide range of therapeutic agents including nano-, microparticles, depot forming gels, or drug solutions [264]. The success of injectable delivery depends on their ability to target the specific anatomic site of action for those active substances allowing sustained delivery in smaller doses. With topical eye drops, for example, more than 95% of the drugs are not absorbed into the eye, and only a small portion enters the ciliary body [274]. MNs have, therefore, emerged as promising strategies for treating not only glaucoma but also other ophthalmic diseases.

Solid MNs form transient microchannels to improve the permeability of active substances or deliver free or encapsulated drugs, peptides, or vaccines. However, solid MNs are made of stainless steel and silicone, which are non-biodegradable. Therefore, further preclinical studies are needed to determine their safety and efficacy for intraocular drug delivery [117,268].

Khandan et al. manufactured fenestrated, titanium MNs for ocular drug delivery with a potential for safe, simple, and reliable application, as well as uniform coating with drug formulations. They assumed that the optimal dose for the administration of pilocarpine for one month would be only 150 μg, indicating a significant dose saving with using MNs [266].

**Figure 4:**
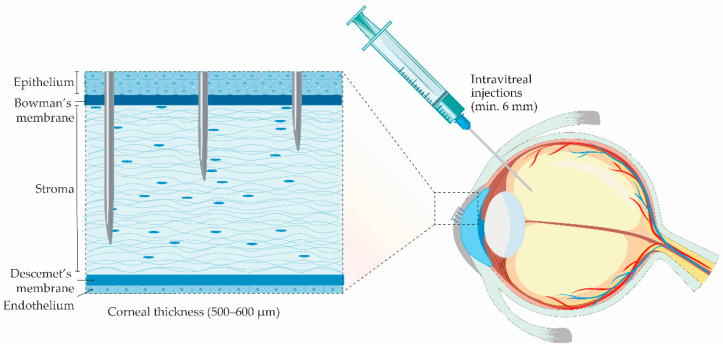
Comparison of ocular administration of intravitreal injection and microneedles (MNs). Due to the MN length and curvature of the corneal, all tissue structures can be reached. In comparison, during intravitreal injection, it is necessary to insert the needle into the vitreous to a depth exceeding 6 mm [276].

More frequently, a solid MN surface is coated with a drug formulation [267]. Their application creates micron-sized pores within the sclera or cornea that enable effective drug delivery and promote the release of active substances located in coating formulation (Figure 4) [268,275].

Jiang et al. reported the delivery of pilocarpine-coated MNs to the anterior segment of the eye. This type of MNs, 500–700 μm in length, increased the absorption rate of pilocarpine by approximately 45 times. The results showed the excellent penetration of MNs into the sclera (up to 300 μm) and a rapid dissolution rate of active substances into the eye. MNs caused fast and extensive constriction of a pupil from 8 to 5.5 mm diameter within 15 min, while the topical delivery of pilocarpine narrowed the pupil to just 7 mm [275].

Hollow MNs allow the rapid delivery of different formulations to a specific site within the eye through the lumen of MNs [268]. Studies have reported a minimally invasive drug delivery by hollow MNs within ocular tissues such as the SCS, sclera tissue, or other ocular tissues [277,278]. Due to the curvature of the cornea, MNs can penetrate in different ways and depths into the eye, thus, providing a complete drug release in all corneal structures [262]. They can be made from borosilicate glass or biodegradable polymer. However, the first one is not suitable for clinical application [268]. Hollow MNs provide drug delivery for the treatment of glaucoma [268].

Jiang et al. made hollow MNs and infused different formulations containing model drug (sulforhodamine), nanoparticles, and microparticles into the scleral tissue. Microneedles were inserted into the tissue at a depth of 700–800 μm, and then the drug solution was infused at a pressure of 103.4 kPa. After the further withdrawal of 200–300 μm, they achieved a successful delivery of 10–35 μL of solution into the sclera, suggesting the formation of an intrascleral drug terminal. However, microparticles were delivered just in case of the presence of hyaluronidase and collagenase spreading enzymes that breakdown the components of the tissue [278].

Chiang et al. fabricated hollow, metal microneedle injections for highly-targeted delivery of brimonidine PLA microspheres to the SCS of the rabbits’ eyes. They reported a 6 mm Hg reduction in IOP and a significant dose sparing due to the increased bioavailability of brimonidine, in comparison to daily administration of eye drops. This approach provides a sustained-release treatment for glaucoma, and injections were well-tolerated without side effects as the drug is located in the SCS away from other non-targeted tissues [279].

Kim et al. fabricated a single, hollow 33-gauge MN (700–800 μm in length) and inserted it into the sclera to inject the antiglaucoma drugs into the supraciliary space of rabbits’ eyes. They reported successful targeted delivery of either sulprostone or brimonidine into the supraciliary space and lowering of IOP by as much as 3 mm Hg. Furthermore, the significant dose sparing (100-fold) in comparison to topical eye drops is reported [280].

The same group investigated the drug delivery to the ciliary body and choroid with hollow MNs, less than 1000 μm in length, through SCS. They have shown the successful delivery of particles up to 10 μm in diameter that can be localized in the SCS, indicating that they can be used for minimally invasive treatment compared to a surgically placed implant. Also, this therapeutic approach offers highly-targeted therapy for glaucoma medications in comparison to intracameral or intravitreal injection [281]. In contrast to intrastromal or intrascleral injection of MNs, Patel et al. demonstrated safe and minimally invasive delivery of sulforhodamine B and nano- and microparticle suspensions (up to 35 μL) into the posterior segment of the eye of a rabbit, pig, and human eyeballs with a single, glass hollow MNs 800–1000 μm in length and applied pressures of 250–300 kPa [282].

To overcome the problems linked to coated and hollow MNs in terms of their fabrication, accuracy, or difficulties in injecting the drug solution, dissolving polymeric MNs, made of biodegradable, biocompatible polymers, were proposed for ocular drug delivery. MNs are first applied to the ocular tissue and then, the active substances incorporated within the polymer matrix are released into the eye [283].

Roy et al. fabricated a dissolving microneedle ocular patch (550 μm in length) to mimic commercially available contact lenses, for delivery of pilocarpine hydrochloride. They concluded that MNs made of PVA and PVP dissolved immediately upon administration and provided a deliver significantly higher flux of pilocarpine compared to pilocarpine solution [284].

Taking everything into consideration, it is noteworthy that MNs have many advantages over an intravitreal injection, which completely penetrates across the sclera, choroid, retina, and into the vitreous body. MNs, on the other hand, penetrate directly into the sclera and avoid complications associated with conventional ophthalmic therapeutic strategies (Figure 4). MNs might also permit the self-administration, and the treatment of glaucoma, in that case, would only require a routine visit to the doctor.

In short, interest is growing in this area, as MNs can be used in a minimally invasive way for the administration of various drug molecules, not just antiglaucoma drugs, both for rapid and possible controlled drug release. Although tissue trauma after MN application is significantly smaller than the hole made by intravitreal injections, it is necessary to conduct further studies so as to obtain additional information on the mechanism and duration of recovery.

Furthermore, more research should be done in the fabrication of MN by low-cost manufacturing methods, suitable for mass production, and developing appropriate devices for the eye. Only when safety studies examine MN application forces, tissue type, penetration depth, formulation types, and appropriate animal model, MNs will overcome all regulatory obstacles. Overall, it seems that MNs will revolutionize the way drug formulations are administered in the eye in the future.

### 3.8. Ocular Iontophoresis

Iontophoresis is a non-invasive process that allows greater drug BA when applied to the anterior or posterior eye segments, compared with the topical application of conventional formulations. This method is also safer than conventional dosing accompanied by systemic absorption or intravitreal injections which can lead to side effects and infection development. The drug delivery is based on the principle of attracted or repulsive charges. This enables an improved penetration of the charged drug through biological membranes and the delivery of positively or negatively charged substances into the target tissue (Figure 3G).

The amount of drug that enters the eye can be regulated in two ways: (1) by the strength of the current, or (2) by the duration of the treatment. In this way, the eye gets a therapeutic drug concentration and at the same time, systemic side effects can be avoided by minimizing systemic absorption [285,286].

The EyeGate II Delivery System (EGDS) developed by Eyegate Pharmaceuticals, Inc., is a novel ocular iontophoresis system that resembles all the above-mentioned advances. It consists of an applicator on the conjunctiva and a generator connected to an electrode attached to a patient’s forehead (Figure 3G).

The generator generates an electric field in the applicator and the opposite charge on the electrode. A drug is located in the applicator. During the period of electrical stimulation, a drug passes through either the cornea or the sclera. The GDS is still in clinical trials, but it is showing promising results in the treatment of anterior segment diseases and great potential for use in posterior segment diseases. Future research will focus in particular on the possibility of using EGDS in potentially blinding posterior segment diseases [287].

## 4. Future Perspectives

### 4.1. Future Perspectives in Wound Healing Modulation

Since various growth factors are involved in wound healing, one of the directions in which the development of anti-scarring drug delivery systems takes its first steps is inhibition of growth factors. Some of these approaches have already been proposed and investigated.

TGF-β is involved in wound healing, especially in scarring not only in the skin but throughout the body. TGF-β2 is its predominant ocular isoform and plays a crucial role in conjunctival scarring, so impairment of its production is one of the targets in the modulation of wound healing [288]. The decorin is a naturally occurring TGF-β inhibitor and, therefore, it is considered safer than clinically tested anti-TGF-β monoclonal antibodies (CAT152) [289], but to the best of our knowledge, no human studies on decorin have yet been conducted [33] but is very attractive to formulate it.

CAT-152 (lerdelimumab) represents a human monoclonal immunoglobin G4 antibody that neutralizes TGF-β2, which means that it may be employed in therapeutic purposes as an inhibitor of scarring. Considering the encouraging results obtained from phase I and phase II clinical studies, a phase III study of CAT-125 on 388 patients with a diagnosis of primary open-angle glaucoma or chronic angle-closure glaucoma was conducted. Subjects were received three doses of subconjunctival injection of CAT-152. The first one was applied before surgery, approximately 1 cm from the limbus. The second and third injections were given immediately after surgery, and the day after, respectively. The results have shown that there were no statistically important differences between the control and placebo groups regarding treatment success (*p* = 0.22294). Furthermore, in patients that received 5-FU for postsurgical management to prevent incipient scarring, treatment success was less likely (*p* = 0.0003). However, a higher rate of successful treatment was found in patients with primary open-angle glaucoma than other groups (*p* = 0.0077) [290].

Inhibition of VEGF also reduces scarring after glaucoma surgery. Animal and human studies have shown that subconjunctival injection of bevacizumab, a monoclonal humanized anti-VEGF antibody, is effective in reducing scar formation [291,292,293,294]. Briefly, Vandewalle et al. performed a one-year randomized study in 138 patients with medically uncontrolled open-angle glaucoma. Patients received a 1.25 mg/0.05 mL of bevacizumab intracamerally to examine if this injection may improve the outcome of the primary trabeculectomy. The results from this study have shown that IOP was significantly lower one year after surgery, but no significant difference between treatment groups was found (*p* = 0.69). However, an absolute success was better in patients that received bevacizumab (*p* = 0.02), indicating that it can be used as an adjuvant therapy to improve surgical outcomes [295]. These results are in correlation with other studies [291,295,296,297], where it was concluded that bevacizumab can be used to reduce the incidence of bleb failure after trabeculectomy. Interestingly, Akkan and Cilsim reported that, during a 12-month follow-up study in 42 patients, better control of IOP at levels below 12 mmHg was achieved when MMC was given topically after primary trabeculectomy compared with subconjunctival administration of bevacizumab. Besides, a higher number of patients from the bevacizumab group required antiglaucoma drugs [298]. Similar results were obtained in a randomized study in 34 patients with uncontrolled glaucoma. Nilforushan et al. compared whether the outcome of trabeculectomy is better when bevacizumab or MMC is used. They reported an IOP reduction of 34 and 56% at six months, in bevacizumab and MMC groups, respectively. However, better control of IOP is achieved with the administration of MMC [299].

However, this field has to be broadened with more studies, which will address problems linked with the duration of action, and the toxicity effects on the corneal endothelium, lens, and trabecular meshwork. It is also necessary to examine if anti-VEGF agents are capable of blocking all VEGF isomorphs at once [300].

Since bevacizumab is only approved as an anti-cancer agent and not yet for use after surgery, this opens a world of possibilities in the effort to develop a drug delivery system with this active that is best suited to the eye. With this in mind, Giannos et al. developed a novel, stabilized formulation that prevents antibody aggregation, suitable for ocular drug delivery. They used NIR light radiation to enhance the permeability of bevacizumab through the sclera and demonstrated that it can deliver clinically relevant amounts of drugs non-invasively via the sclera during a one-hour treatment [301].

On the other hand, Won et al. developed 3D printed multi-shell rods, using a coaxial printing technique, for the simultaneous delivery of bevacizumab and dexamethasone. The developed rod consisted of a PCL-bevacizumab shell and an alginate-dexamethasone core fabricated using a multiple-head 3D bioprinter. This system provided sustained release of bevacizumab for 50 days, after the initial burst release within 3 days. Dexamethasone showed burst release within 24 h with subsequent undetectable release amounts. This rod showed better short- and long-term therapeutic efficacy compared to commercial intravitreal injections [302].

Future perspectives in wound healing include interference with various cytoskeletal regulators (Rho-kinase inhibitors, paclitaxel), growth factors (inhibitors of TGF-β, VEGF), cytokines (interferon-α), and proteinases (matrix metalloproteinases) [29,33,303]. It remains to be seen what formulation solutions researchers will offer to fight scarring after glaucoma surgery.

### 4.2. Gene Therapy

Through the advances of genetics and bioengineering that enabled manipulating vectors for delivery of extrachromosomal material to target cells, gene therapy has emerged as a new strategy that has been investigated extensively. The main focus of this novel therapy is the correction or inactivation of mutated genes, that are responsible for causing a certain disease, through the insertion of a normal gene into the genome [304]. Furthermore, genome editing techniques such as CRISPR/Cas9 have opened new therapeutic opportunities [305].

Taking into account that glaucoma is a chronic disease, promising new therapeutic strategies such as gene therapy that can target and correct relevant pathophysiology can be used. Gene therapy has the potential to provide a long-lasting IOP lowering effect through increasing conventional outflow, increasing uveoscleral outflow, or decreasing aqueous humor production. Slowing or preventing neuronal death through the expression of neuroprotective agents is also another important approach of gene therapy in glaucoma [306].

In order to identify gene therapeutic targets, the genetic basis of glaucoma must be well investigated and understood. Researchers discover at least 29 genetic loci for various forms of glaucoma, and 12 causative genes have been identified from these loci [307].

Potential vectors suitable for glaucoma gene therapy are both viral and non-viral vector delivery systems. Non-viral vectors are characterized by low toxicity and immunogenicity. However, they are generally less efficient in gene delivery and achieve lower transfection efficiency and lower production scales compared to virus-based vectors. Viral delivery systems include herpes simplex viruses, adenovirus, adeno-associated viruses, and lentiviruses. Among other viral vectors, recombinant adeno-associated viral vectors (AAVs) have proven the most promising [308]. Non-viral gene delivery systems include liposomes/synthetic polymers, direct injection of naked DNA into tissues, RNA interference, and electroporation [306].

Reduction of cellular contractility through inhibition of Rho kinase (Rho GTPase) can increase outflow facility in organ cultured porcine and human eyes and in monkey eyes in vivo. Different genes (DN Rho, C3, DNRK, Caldesmon) were studied in order to manipulate this Rho kinase pathway that plays an important role in the modulation of aqueous humor outflow through the expression of protein inhibitors of this cascade [309,310,311]. In their research, Vittitow et al. showed that inactivation of RhoA by adenoviral gene transfer to the intact human outflow pathway increases outflow facility. They suggested that adenoviral vectors carrying the dominant-negative form of RhoA could potentially be utilized as gene therapy to modulate outflow facility [312].

Gabelt et al. concluded in their study that overexpression of an endogenous protein (caldesmon) via gene transfer can be used to modulate outflow facility in the primate trabecular meshwork (TM) in vitro [311].

Gene therapy may also be used to increase uveoscleral outflow, by enhancing or suppressing the endogenous targets that are ultimately responsible for the outflow enhancement triggered by MMPs and tissue inhibitors of the MMPs (TIMPs).

A novel approach for the treatment of steroid-induced glaucoma was introduced by Spiga et al. They constructed a novel glucocorticoid-inducible adenovirus vector that overproduces MMP1 only in the presence of dexamethasone [313]. Results obtained by Kee et al. also indicate the possibility of treating glaucoma by expressing the stromelysin gene within the uveoscleral outflow pathway [314].

Inhibition of myocilin gene (MYOC), by RNA interference approach using synthetic small interfering RNAs (siRNAs) and short hairpin RNAs (shRNAs), was also investigated because primary open-angle glaucoma-associated mutations have been found in myocilin gene.

Comes et al. delivered naked siRNAs to the intact human TM by intracameral perfusion in order to inhibit the MYOC gene and their results revealed a successful decrease of MYOC secreted by siRNA-treated cell and organ cultures [315]. An interesting approach was recently introduced by Jain et al. who used CRISPR-Cas9–mediated genome editing in cultured human TM cells and in an MYOC mouse model of primary open-angle glaucoma to knockdown expression of mutant MYOC which results in lower IOP and prevents further glaucomatous damage [316].

Wu et al. used a pragmatic gene therapy approach in their study, that reduces IOP by selectively disrupting aqueous humor production by disrupting Aquaporin 1 (Aqp1) in the ciliary body. They used single recombinant adeno-associated virus (ShH10) to deliver *S. aureus*-derived CRISPR-Cas9 platform to disrupt Aqp1 which resulted in reduced IOP in treated eyes (10.4 ± 2.4 mmHg) compared with control (13.2 ± 2.0 mmHg) or non-injected eyes (13.1 ± 2.8 mmHg; *p* < 0.001; *n* = 12) [317].

Using siRNAs that target the carbonic anhydrase gene IV and the b2-adrenergic receptor resulted in decreased intraocular pressure in a rabbit model of ocular hypertension by inhibition of aqueous humor secretion [318].

The use of siRNA against the β2-adrenergic receptor (ADRβ2) could also provide an interesting therapeutic strategy for glaucoma treatment. SYL040012, siRNA targeting ADRβ2 is currently under development for glaucoma treatment. After topical ocular instillation in animal models, SYL040012 specifically inhibits the synthesis of the β2-adrenergic receptor (ADRβ2) which leads to IOP reduction [319]. In the phase I clinical trial, which was performed in healthy volunteers with normal IOP values (<21 mmHg), SYL040012 eye drops showed good safety and tolerability at the maximal dose used [320]. SYL040012 is currently undergoing Phase 2 clinical trials [321].

Recently, Pfeiffer et al. used the intravitreal injection of ISTH0036 (modified 14-merfully phosphorothioate antisense oligonucleotide), which selectively targets the transforming growth factor-beta 2 (TGF-β2), which has been linked to the main pathophysiologic events in glaucoma. The conducted study on patients with primary open-angle glaucoma showed that single-dose ISTH0036 administration, at the end of trabeculectomy, is safe and resulted in IOP values persistently below 10 mmHg over the three months postoperative observation period [322].

Unlike most forms of early-onset glaucoma which usually has a single genetic cause, adult-onset glaucoma has an unclear, heterogeneous etiology involving multiple genetic factors, individual risk factors, and environmental factors which complicates standard gene therapy approaches. Thus, gene therapies for adult-onset glaucoma have focused primarily on neuroprotection which is based on slowing the loss of retinal ganglion cells (RGC) or by inhibiting the progression of cell death [308]. Studies with intraocular delivery of the genes for neurotrophic factors (such as brain-derived neurotrophic factor (BDNF), ciliary derived neurotrophic factor (CNTF), and neurotrophin-4 (NT-4)) remain encouraging [323,324,325,326,327].

In 2020, Petrova et al. published a very important study in which they demonstrate robust axon regeneration in the adult CNS driven by overexpression of the adapter protein Protrudin. Expressing the Protrudin mRNA (Zfyve27) which is normally found at low levels in nonregenerative neurons, promotes regeneration in the adult CNS by functioning as a scaffold to link axonal organelles, motors, and membranes. This can be very important in gene therapy for glaucoma as Protrudin expression can also promote RGC regeneration. In the current research, an optic nerve crush model, as well as an acute retinal explant model and adeno-associated virus (AAV) delivery vector, were used to examine the effects of Protrudin expression on RGC neuroprotection. Results indicated that both phosphomimetic and wild-type Protrudin led to a large number of axons regenerating for a long-distance only two weeks after optic nerve crush with only phosphomimetic Protrudin having a pronounced effect on neuronal survival 2 weeks post crush. Results also suggest that both forms of Protrudin, were completely neuroprotective in a retinal explant model of RGC injury and two weeks post optic nerve crush [328].

We believe this promoting avenue of research will replace in major part other traditional glaucoma therapy methods. Nevertheless, significant challenges remain before gene therapy can be used to treat glaucoma. A better understanding of the molecular pathogenesis and genetic basis of a disease, optimization of vector systems to improve transfection efficiency and achieving long, sustained levels of therapeutic gene expression within a select target cell, and establishment of more suitable animal models will help realize the potential clinical applications of this promising new therapeutic strategy.

### 4.3. Neuroprotection

Neuroprotection is a treatment independent of intraocular pressure reduction which prevents the death of retinal ganglion cells, thus stopping or delaying blindness [329]. The death of retinal ganglion cells leads to visual field loss. Neuroprotective drugs are more effective in conditions where neuron death is slow than in conditions where neurons die quickly. One of the glaucoma characteristics is the slow death of neurons, so neuroprotection may result in slower and reduced loss of ganglion cells. The benefits of using neuroprotective drugs as part of glaucoma treatment are greater when the corresponding amount of drugs is administered to the retina and the drug has few side effects [330]. Neuroprotection strategies are based on two approaches: using signaling pathways to stimulate cell survival or inhibiting cell death pathways to increase the ability of cells to withstand pathological insult [331]. The challenges in neuroprotection for glaucoma are diverse, including the development of adequate animal models that correspond to the human disease. While animal models of neurodegeneration have been developed, only a few approaches have been able to be translated into clinical trials [332].

#### 4.3.1. Glutamate Antagonists

The excitotoxic effect of glutamate in the retina occurs due to the high extracellular glutamate level. The glutamate interacts with glutamate excitatory receptors in RGCs and causes their overexpression. Glutamate accumulation overstimulates *N*-methyl-d-aspartate (NMDA) receptors. As a result, the influx of calcium ions results in the production of free radicals, which ultimately leads to apoptosis. By modulating NMDA receptors, glutamate activity can be reduced [333]. Dreyer et al. showed a double increase in glutamate levels in the vitreous body of glaucoma patients, indicating the possibility of an excitotoxic mechanism leading to the death of RGCs [334].

MK801, also known as dizocilpine maleate is an uncompetitive NMDA antagonist and is considered the most potent glutamate inhibitor and neuroprotective agent in the experimental treatment of glaucoma. Due to its long half-life, interruption of normal physiological functions of glutamate, and thus neurotoxicity, it has never been evaluated in higher-level clinical trials [335].

Memantine is a non-competitive NMDA receptor antagonist used in the treatment of moderate to severe Alzheimer’s disease and has shown promising results in a monkey model of glaucoma [336]. NMDA receptors are widespread throughout the central nervous system and are critical for neurotransmission and the healthy function of neuronal cells. However, overstimulation in the presence of excessive glutamate can lead to Ca^2+^-mediated neurotoxicity. Dysregulation of this cascade has been widely implicated in many chronic neurodegenerative conditions including glaucoma [331].

Bis(7)-tacrine is a newer NMDA receptor antagonist with exceptional neuroprotective activity. It inhibits acetylcholinesterase and nitric oxide synthase and consequently blocks the NMDA receptor [337]. Bis(7)-tacrine demonstrated a more potent neuroprotective effect than memantine in a study on cultured RGCs [338]. Amantadine, psychotropic tetrahydrocannabinol, and non-psychotropic cannabinol achieve their neuroprotective effects by reducing the NDMA activity [339,340].

#### 4.3.2. Neurotrophic Factors

The lack of neurotrophic factors in the optic nerve contributes to the occurrence and progression of glaucomatous optic neuropathy (GON) which is caused by high intraocular pressure (IOP). Improvement of neurotrophic support may delay the loss of RGCs in glaucoma [341]. In glial cells and neurons exist receptors for various neurotrophic factors. The absence of neurotrophic factors leads to blockage of axonal transport and eventually apoptosis [342].

Brain-derived neurotrophic factor (BDNF) belongs to a group of growth factor proteins. It is of great importance for the function and survival of RGCs. It effectively prevents lesion-induced axonal death in the rat optic nerve, but can’t prevent the rapidly progressive degeneration of RGCs after axotomy [343].

The main disadvantage of intravitreal injections of BDNF is their inability to deliver the protein continuously and the need for repeated administration. To overcome this, other novel approaches have been developed through the use of gene therapy or stem cells [341].

Ciliary neurotrophic factor (CNTF) also showed a neurotrophic effect on RGCs. Intravitreal administration of CNTF can protect RGCs from apoptosis. Pease et al. overexpressed CNTF in the retina of Wistar rats with chronic ocular hypertension to investigate its protective effects on RGCs. Exactly 15% less RGCS axon loss was observed in the eyes that received intravitreal injections of CNTF-AAV vectors [344].

The GDNF-loaded biodegradable microspheres were studied by Jiang et al. to determine the efficacy of this trophic factor. GDNF microspheres were injected intravitreally and the treatment demonstrated effective neuroprotective effects on RGCs in Brown Norway rats with chronic ocular hypertension, such as increased number of RGCs axons, the thickness of the retinal inner plexiform layer (IPL), and level of glial fibrillary acidic protein expression. Advantages of microspheres used in this study included continuous delivery of the trophic factor and economic feasibility [345].

Artemin, basic fibroblast growth factor, interleukin-6, and erythropoietin are other trophic factors or cytokines for which a neuroprotective effect has been suggested [333]. Effective and sustained delivery of trophic factors to the retina is challenging because of the large molecules of these proteins whose passing through the blood-retina barrier can be difficult. An alternative delivery route of trophic factors to the retina could be intravitreal injection but this route of administration is not convenient in chronic conditions such as glaucoma. The integration of neurotrophic factors in drug delivery devices for intraocular implantation is a possible approach for the long-term provision of such agents. Although viral vector-delivery of trophic factors has shown protective effects in animal models of retinal degeneration, certain issues such as precise dosage control call into question the clinical application of this approach [346].

#### 4.3.3. Antioxidants

Tissue samples and aqueous humor samples from eyes with glaucoma show lower antioxidant levels increased oxidative stress markers, antibodies against glutathione-S-transferase decreased plasma levels of glutathione, increased lipid peroxidation products in the plasma, higher concentrations of reactive oxygen species (ROS) [347], decreased cell membrane potentials and decreased ATP production in the trabecular meshwork (TM). Therefore, oxidative stress leads to the death of RGCs by injuring the optic nerve head and trabecular meshwork. Antioxidants can neutralize ROS and prevent their accumulation in TM [348,349]. Cell defense mechanisms against oxidative stress include superoxide dismutase, glutathione (GSH), and thioredoxin (TRX) systems [350].

Vitamin E deficiency can increase levels of lipid peroxidation products and lead to RGCs death. Subcutaneous application of vitamin E can prevent retinal injuries caused by high IOP [351].

Nicotinamide (vitamin B3) can also be used as a neuroprotective agent. NAD is an essential co-factor in the redox reactions of the mitochondrial respiratory chain required to maintain sufficient levels of adenosine triphosphate (ATP). The intraocular part of the retinal ganglion cell axon is unmyelinated and therefore has a particularly high energy requirement. Nicotinamide deficiency and NAD depletion, therefore, have the potential to disrupt mitochondrial and energy metabolism as well as the function of the ganglion cells [331].

Coenzyme Q10 (CoQ10) stabilizes the mitochondrial membrane potential, supports ATP synthesis, and inhibits the generation of ROS. It also prevents retinal damage caused by transient ischemic injury due to acutely elevated IOP. CoQ10 inhibits mitochondrial depolarization by preventing the formation of the mitochondrial permeability transition pore thus protecting the RGCs from glutamate excitotoxicity in vivo. It may reduce the accumulation of extracellular glutamate and thereby reducing the harmful effect of ischemia/reperfusion on mitochondrial energy metabolism and glutamate transporter function, thereby preventing the apoptotic death of RGCS [352].

Topical treatments with CoQ10 or vitamin E in established rat models of glaucoma showed their effectiveness in preventing RGCs apoptosis. Systemic administration of CoQ10 in DBA/2J mice preserved the axons in the optic nerve head by inhibiting oxidative stress, as demonstrated by suppressed superoxide dismutase-2 (SOD2) and hem oxygenase-1 protein expression [353].

Alpha-lipoic acid and SOD are the other two essential antioxidants. Nebbioso et al. concluded that 8 weeks of treatment with alpha-lipoic acid and SOD provide RGCs protection from damage induced by acute ocular hypertension [354].

Melatonin can be used as a neuroprotective agent due to its antioxidant, anti-excitotoxic, and anti-inflammatory activity. It can cross the blood-brain barrier and also has a short half-life and no significant side effects [355]. Bessone et al. developed an innovative nanometric system for the controlled topic release of melatonin in the retina. Ethylcellulose nanocapsules were characterized by various physicochemical techniques and investigated in ex vivo and in vitro studies on albino rabbits. In vitro release of melatonin was slower compared to a melatonin solution. However, this system caused greater penetration and no irritation during the transcorneal application. Melatonin included in ethylcellulose nanoparticles had enhanced neuroprotective effects on RGCs [356].

Citicoline (cytidine 5′-diphosphocholine) shows a possible protective effect on RGCs due to its antiapoptotic effect in mitochondrial-dependent cell death mechanism. It also shows an auxiliary role in axon regeneration [357]. Parisi et al. discovered that the neuroprotective effect of citicoline in form of eye drops is independent of the IOP-lowering effect and also confirmed that the administration of citicoline eye drops significantly improved pattern electroretinogram (PERG) and visual evoked potential (VEP) [358].

Administration of palmitoylethanolamide inhibits various inflammatory cascades relevant to glaucoma and shows the ability to lower IOP and improve visual field indices in glaucoma patients with normal-tension glaucoma, indicating a dual IOP-lowering and neuroprotective effect [359].

Other antioxidants such as vitamin C, α-tocopherol, and ginkgo biloba can also be used to prevent RGCs damage. Polyphenolic flavonoids from ginkgo are capable of penetrating into the mitochondria and therefore can be beneficial as a neuroprotective agent due to the maintenance of mitochondrial metabolism. Conversely, an extract of ginkgo can increase the risk of bleeding during surgery so it should be administrated with caution [329].

#### 4.3.4. Calcium Channel Blockers

The neurotoxic effect of NMDA is mediated by calcium influx into neural cells, followed by apoptosis and cell death. Calcium-channel blockers (CCBs) can prevent RGCs death caused by calcium influx. Additionally, they improve local blood flow in ischemic tissues by inducing vasodilation. Various calcium channel blockers such as iganidipine, nimodipine, and lomerizine have been shown to significantly increase the viability of purified rat RGCs under hypoxia [360]. Koseki et al. reported that in a placebo-controlled study conducted in primary open-angle glaucoma (POAG) patients, oral nilvadipine for 3 years slightly slowed visual field progression and maintained the optic disc rim [361]. In another laboratory study, diltiazem could not prevent glutamate-induced RGCs apoptosis in contrast to nilvadipine [362]. Patients taking CCBs may experience reduced blood flow, which may interrupt the autoregulation of blood circulation and accelerate pathological changes in RGCs. Reduction of RGCs loss and thinning of the inner retinal layer is accomplished with an intraperitoneal injection of flunarizine in a rat model of acute IOP. Pretreatment with lomerizine, a specific blocker for L- and T-type Ca^2+^ channels, showed protective effects against glutamate-induced excitotoxicity in rat retinal cell cultures [363,364].

#### 4.3.5. Alpha 2 Adrenergic Agonists

Brimonidine is a widely used ocular hypotensive by activation of α_2_ adrenergic receptor and decrease in aqueous humor production. Its neuroprotective effects are independent of its effect on IOP. These effects are mediated via a variety of mechanisms including brain-derived neurotrophic factor (BDNF) and basic fibroblast growth factor upregulation, nitric oxide synthase 3 metabolism and retinal vasomodulation, the activation of cell survival signaling pathways and prevention of apoptosis and modulation of NMDA receptor function [331]. If brimonidine is administrated intravitreally instead of topically, RGCs survival can be significantly improved through upregulating endogenous expression of BDNF in RGCs [365]. Based on a systematic review and meta-analysis of evidence on the neuroprotective properties of brimonidine in glaucoma, Scuteri et al. reported that assessing the efficacy of brimonidine on visual field deterioration during glaucoma should be further investigated and that its neuroprotective effects are inconclusive and need stronger support, such as large double-blind randomized clinical trials [366].

#### 4.3.6. Nitric Oxide Synthase Inhibitors

A form of nitric oxide synthase (NOS), NOS-2 exhibits neurotoxic effects [367]. The inhibitory effects of aminoguanidine and *N*-nitro-l-arginine on NOS-2 were confirmed in neuroprotection and delayed RGCs degeneration [368]. In a study conducted by Neufeld, oral administration or topical application of aminoguanidine in rat models with either chronic or acute IOP elevation significantly enhanced RGCs survival. Another NOS-2 inhibitor, SC-51, had similar protective effects on RGCs when used in a Brown Norway rat model with chronic high IOP [369]. However, some studies displayed no significant changes in NOS-2 expression and no effect of aminoguanidine treatment on glaucomatous damage in rats [370].

#### 4.3.7. Anti-Glaucoma Medications with Blood Regulation Effect

Improved regulation of ocular blood perfusion can subsequently provide better neuroprotection. Drugs that have been studied for these purposes are CAIs, latanoprost, and betaxolol [371]. Topical application of betaxolol in SD rats with acute ocular hypertension preserved the integrity of the inner plexiform and nuclear retinal layer. Collignon-Brach reported that patients with ocular hypertension or chronic open-angle glaucoma received visual field benefits after taking betaxolol for three years, although timolol lowered the IOP more effectively [372].

#### 4.3.8. Heat Shock Proteins

Heat shock proteins (HSPs), also known as stress proteins or molecular chaperones are present in numerous cells. They are necessary for the normal function of cell proteins and they impede the apoptotic pathways. They can prevent cell death both directly (by inhibiting proapoptotic factors) and indirectly (by protecting from oxidative stress and suppression of proinflammatory cytokines) [373]. Piri et al. described the essential role of HSPs in RGCs survival. When the eye is exposed to heat or radiation, the reaction of ocular stress is triggered and the level of HSPs increases. Heat shock factors (HSF) are located in the nucleus and bind to heat shock elements (HSE) to form a complex structure in the promoter area of the selected gene. This leads to the transcription of HSPs and the stress response is primarily regulated by HSF at the transcriptional level [374].

#### 4.3.9. Stem Cell Transplantation

Stem cells can produce new cells of all kinds and induce the regeneration of the damaged ones. The supply of various neurotrophic factors is the most widely accepted mechanism by which transplanted cells can modulate excitotoxicity. One of the benefits of stem cell transplantation for RGCs neuroprotection is its long-lasting and localized effect which is intended to improve patient compliance. Long term safety trials are essential to ensure that the potential benefits of neuroprotection outweigh the risk of tumor triggering [375].

Unlike the peripheral nervous system (PNS), the central nervous system (CNS) does not possess the property of self-regeneration. Stem cells’ transformation into healthy RGCs requires their integration into the retina, directing them to sprout new axons that grow out of the eye and into the optic nerve and the formation of new and appropriate synapses by reaching their synaptic target in the diencephalon. All of this is very difficult to achieve [376].

RGCs regeneration can be accomplished with various cells, such as embryonic stem cells (ESCs), induced pluripotent stem cells (iPSCs), mesenchymal stem cells (MSCs), human embryonic stem cells (hESCs), and retinal progenitor cells (RPCs) [377].

Three-dimensional (3D) retinal organoids are in vitro tissue structures derived from self-organizing cultures of differentiating hESCs or iPSCs [378]. They recapitulate many aspects of retinal development in vitro, expressing the same sequence of transcription factors that characterize endogenous retinal development and self-organizing into layers similar to the native retina. Large amounts of immune compatible, healthy RGCs can be purified from iPSC organoids and transplanted into patients because iPSC-derived retinal organoids develop functional, albeit basic, retinal circuit [379]. In combination with genome editing tools, 3D retinal organoids could become a reliable and renewable source of transplantable cells for personalized therapies for diseases caused by malfunction or degeneration of photoreceptors or RGCs [378].

Stem cells transplantation has been extensively researched as a therapeutic option to replace the human retina inability to regenerate damaged photoreceptors (PRs) or retinal pigmented epithelium (RPE). Photoreceptors derived from retinal organoids have great potential for therapeutic photoreceptor transplantation. Stem cell-derived photoreceptors can be further developed, for example, by expressing optogenetic tools to study functional integration into the host retina [380]. While newly integrated photoreceptors require only short connections to the underlying horizontal and bipolar cells, RGCs need to elongate long axons and restore functional connections to their specific brain targets [381].

Transplantation of RGCs showed some functional recovery of the retina from NMDA-induced RGCs damage. The observed functional rescue can be explained by neuroprotective effects. So far, the only successful transplants of RGCs, leading to the integration of electrophysiologically functional neurons have been described for primary RGCs, with an overall success rate of only 10% [382,383].

Due to these challenges, RGC transplantation compared to RPE or PR transplantation is still at an early stage of the preclinical study. Host development is critical for successful cell replacement in glaucoma patients, but the cause for that is still unclear. Comparing stem cell-derived RGCs and primary RGCs in the early postnatal stage would be valuable in identifying cell stage-specific parameters that promote integration into the mature retina. Despite successful engraftment in some cases, studies have not yet shown any apparent functional improvement after RGC transplantation, due to incomplete cell integration and axon growth [384].

Due to the complex morphology of the neural retina and its various cell types connected to multiple synaptic junctions, transplantation of a single cell type is normally not sufficient to repair the progressive retinal degeneration. A possible solution to this problem is the production of neural retinal tissue from pluripotent cells in vitro. Meyer et al. attempted to create an optical vesicle-like structure containing rhodopsin positive cells induced in a 2D culture system [385]. Eiraku et al. generated a well-organized optic cup structure from a 3D culture system. In the floating retinal organoids, the three neural retinal layers exist in layers similar to those of the retina in vivo, and the photoreceptors in the organoid are more mature than those in a 2D culture system [386]. MSCs derived from adipose tissue and bone marrow have been proposed as a potential cell source for transplantation in degenerative animal models of the RGCs due to their neuroprotective effect. Following MSCs transplantation, an increase in the number of RGCs and RGCs axons, ONL cells preservation, prolonged survival of PRs, increased secretion of NTFs, immunomodulation, and neurovascularization have been achieved [387]. MSC mechanisms of action may include the upregulation of trophic factors, such as FGF-2, and the modulation of neuroinflammation by increasing the expression of cytokines such as IL-1β in damaged tissue [388]. Organoid-derived RGCs may help to elucidate factors that promote axonal outgrowth [389]. Eastlake et al. demonstrated the ability of Müller glia cells isolated from retinal organoids formed by hiPSCs to partially restore visual function in NMDA depleted RGCs in rats [390].

The potential therapeutic value of RGCs derived from retinal organoids has yet to be thoroughly investigated. There are challenges in defining the adjustable delivery route for effective retinal organoid transfer into the host retina, examining how foreign cell injection affects the native retinal environment, and determining whether donor RGCs integrate and form functional circuits that restore visual processing. Vision restoration requires transplanted RGCs to grow axons that extend beyond the optic nerve head, remyelination by oligodendrocytes, and correct guidance to terminals in the brain where new synaptic formation can occur [391]. Overcoming these hurdles could make stem cell-derived RGCs or RGC precursors convenient and preferred for the treatment of patients with glaucoma.

Despite the successful results of neuroprotective drugs in glaucoma treatment, their major disadvantage is that there is no adequate drug delivery route. Patients show poor compliance with topically applied treatments. Arranz-Romera et al. developed multi-loaded PLGA microspheres into which they incorporated a combination of three neuroprotective agents (melatonin, coenzyme Q10, and dexamethasone). Their goal was to develop a novel sustained-release intraocular drug delivery system for the treatment of glaucoma. By adjusting particle size and shape for their effective incorporation in intravitreal injection, they achieved co-delivery of the encapsulated active compounds in a sustained manner over 30-days with low burst release. A significant improvement in neuroprotective effects was observed compared to empty microspheres or microspheres with only one drug, implying that these multi-loaded PLGA microspheres represent promising solutions in the treatment of glaucoma and other neurodegenerative diseases [392].

Most of the neuroprotective drugs have shown their effectiveness in animal models and these results are not always applicable to humans. Their potential to prevent the RCGs damage is undisputed but further clinical trials are needed to establish accurate and effective treatment methods.

## 5. Conclusions

As is can be seen from the presented results, glaucoma is recognized as a very serious and threatening disease. However, researchers and scientists face numerous obstacles to developing improved novel drug delivery systems and solutions that sometimes look like an art.

Research and development of novel drug delivery systems fighting glaucoma is thriving to achieve three key characteristics: (1) efficacy that is at least equal to or preferably better than what is currently achievable conventional eye drops, (2) risks that are low and acceptable with repeated administration, and (3) third-party reimbursement claims for the drug product [393].

As it turns out, the most intensive research on IGS has been carried out as it is a non-invasive type of treatment, and its form of eye drops is not unknown to the patient. As already mentioned, the main obstacle to topical ocular formulation is its removal by tears and gravity. The first logical approach was to try to increase the viscosity, as this will lead to a longer retention time. Smart polymers proved to be a promising solution, but they also come up against their obstacles. As an example, the high concentration of poloxamers needed for gel transition has been solved by adding other polymers as viscosity enhancers. Although different approaches have been used in the formulation to resolve obstacle throughout increasing viscosity, it should be noted that there is a debate about viscosity or mucoadhesiveness itself is sufficient to ensure improved BA. There is an approach to using other polymers that yield strong gel at lower concentrations. The obstacles in the formulations are sometimes the drugs themselves, as timolol is known for its systemic absorption and therefore its systemic side effects, which must be avoided.

It seems that IGS can be recommended as most convenient to the patients as they are noninvasive and come in the form of are self-administered that are familiar to patients, while allowing for a reduced dosing frequency, e.g., once a day, which can lead to better adherence to therapy. However, they do not extend the IOP reduction for a long time, compared to others. On the other hand, there are somewhat invasive systems such as PPs and MNs, but their convenience to the patient, painless insertion, and prolonged drug release can even put them on the number one of the list of recommendations. In addition, there are commercially available PPs and MNs that can be used successfully. However, they need to be administered by a professional. In terms of efficacy, iDose can be recommended as it had the longest IOP reduction of 12 months. However, the main drawback is that it has to be implanted and removed surgically.

Although much work is being done to develop novel drug delivery systems, for the reasons already mentioned and well known, only a few of them have reached the marked, due to mentioned and well-known reasons. However, researchers and scientists have just received a new incentive to continue their work. In the field of glaucoma therapy, many new perspectives play a role, including nanosystems in the first, which look very promising, but one must not forget to mention a great perspective involving gene therapy and neuroprotective drugs.

When it comes to nanosystems, most intensive research has been conducted on nanoparticles. None of the formulations has yet reached clinical trials, but according to in vivo testing in rabbits, a large number of them look very promising as a novel option for glaucoma treatment. Nano-formulation with the best IOP reduction results to date, which last up to 21 days in vivo in rabbits are gelatin-coated mesoporous silica nanoparticles containing pilocarpine, size 50 nm. Apart from that, SLNs are very interesting and exciting innovations, but those that were able to achieve greater IOP reduction were unable to sustain this effect for more than 8 h. In contrast to the above-mentioned formulations, which induced a moderate IOP reduction, cationic SLNs, size 150–300 nm with melatonin, were able to successfully maintain IOP reduction for 24 h. However, the obstacle posed by the burst release has yet to be resolved. Liposomes in glaucoma treatment have also been extensively researched and must be positively or neutrally charged to ensure a longer corneal residence time and thus a longer IOP reduction. The only nano-formulation administered to humans (*n* = 6) subconjunctivally was that with latanoprost which induced a three-month IOP reduction, which was longer than with all other liposome formulations.

Most of the article focused on approved IOP lowering drugs formulated as a novel, sustained drug delivery systems fighting glaucoma. However, it is important to outline that there are increasingly obvious trends to develop novel drug delivery systems that allow the delivery of anti-scarring and neuroprotective drugs, as well as to develop new therapeutic strategies that include gene therapy that provide a long-lasting IOP lowering effect and/or prevent the death of ganglion cells by expression of neuroprotective agents. In addition to already approved dexamethasone implants that intervene in scarring, new alternatives such as anti-VEGF and anti-TGF-β2 agents, either alone or in combination with conventional therapies, are at different stages of development. It is only a matter of time before a suitable alternative comes onto the market. Apart from this, research is underway to sustain the delivery of neuroprotective drugs to the back of the eye by intravitreal injections. Once the benefits of neuroprotective drugs are proven and established on the market, more activity in sustaining the delivery of such drugs is expected to improve outcomes in glaucoma patients.

## Data Availability

Not applicable.
